# Dual-Expert Landmark Localization in 3D Facial Point Clouds Under Controlled Synthetic Local Surface Loss for Rigid Initialization

**DOI:** 10.3390/s26154989

**Published:** 2026-08-06

**Authors:** Zichao Zou, Zongjian Chen, Rongqian Yang, Kehai Peng, Shizhong Jiang

**Affiliations:** 1 College of Medical Information Engineering, Guangdong Pharmaceutical University, Waihuan East Road, Guangzhou 510006, China; 2112445026@stu.gdpu.edu.cn (Z.Z.); 2112445006@stu.gdpu.edu.cn (Z.C.); 2 School of Electronic and Information Engineering, South China University of Technology, Wushan Road, Guangzhou 510640, China; rqyang@scut.edu.cn; 3 Department of Research and Development, Guangzhou Aimooe Technology Co., Ltd., Kaitai Road, Guangzhou 510535, China; higger@foxmail.com

**Keywords:** 3D facial landmark localization, incomplete point cloud, controlled synthetic surface loss, dual-expert learning, automatic reliability gating, rigid initialization, point-cloud registration

## Abstract

Local surface loss can destabilize landmark-based rigid initialization in three-dimensional (3D) facial point clouds. We propose a dual-expert framework for localizing five anatomical landmarks under controlled synthetic surface loss. The Clean expert is optimized for peak-based localization on complete surfaces, whereas the Occlusion expert combines local coordinate regression, visibility estimation, heteroscedastic modeling, and a global structural prior. A model-output reliability gate removes dependence on the protocol-supplied surface-loss ratio. For scenes not classified as reliably complete, the Occlusion expert provides the default and fallback prediction, while a validation-selected Clean residual is applied only under landmark-wise agreement. On a subject-independent FaceScape test set, mean localization errors were 0.470±0.688, 1.159±1.093, and 2.216±2.169 mm at 0%, 30%, and 50% surface loss. The method significantly outperformed the Unified occlusion-aware and structure-robust (OASR) model and the Occlusion expert at 30% and 50% loss after Holm correction and achieved lower error than Unified OASR in 22 of 24 structured-corruption conditions. In a controlled large-pose stress test, initialization achieved 100% iterative closest point (ICP) success versus 98.82% for the two-stage stratified graph convolutional network (2S-SGCN) baseline and improved coarse alignment, whereas post-ICP accuracy did not differ significantly. These results support robust rigid initialization under controlled synthetic surface loss on FaceScape; generalization to real sensor-acquired point clouds remains to be established.

## 1. Introduction

Three-dimensional (3D) facial point clouds are used in biometric measurement, surface assessment, human–computer interaction, and registration workflows. Structured-light and red–green–blue and depth (RGB-D) systems can provide dense geometry, but shadowed regions, missing-depth areas, non-uniform sampling, range-dependent noise, and view-dependent acquisition effects may produce locally incomplete or degraded surfaces [1,2,3,4,5,6]. Smartphone-centered acquisition has also emerged as a low-cost route to 3D facial geometry. Xie et al. introduced SCIFI, which combines a smartphone front camera with controlled screen illumination to recover facial shape, illustrating how integrated display–camera hardware can support mobile 3D face reconstruction [7]. Sparse anatomical landmarks can provide semantic correspondences for estimating an initial rigid transformation before dense surface refinement [8,9,10,11,12]. Reliable initialization remains important in image-guided and sensor-assisted workflows because point-cloud registration depends on correspondence quality and because local optimization methods such as iterative closest point (ICP) are sensitive to initialization [13,14,15,16,17,18]. Learning-based registration methods have further emphasized correspondence learning and low-overlap matching, including Deep Closest Point and PREDATOR [19,20]; however, these methods address pairwise surface registration rather than sparse anatomical landmark initialization.

Current 3D facial landmark detectors perform well on complete surfaces. FaceScape provides high-quality 3D facial meshes for controlled evaluation [21]. PointNet introduced direct permutation-invariant learning on point sets [22], PointNet++ added hierarchical local aggregation [23], and Point Transformer demonstrated self-attention for point-cloud feature learning [24]. Graph-based heatmap models subsequently improved 3D facial landmark prediction through local and global aggregation [25,26]. PointSurFace further demonstrated the value of local triangular surface descriptors for facial point clouds [27]. Occlusion-robust landmark studies have shown that feature fusion, structural-relation reasoning, and adaptive learning can improve predictions when local facial evidence is unavailable [28,29,30]. However, a complete surface and a surface with missing target neighborhoods do not necessarily share the same optimal localization mechanism.

A dual-expert system that does not receive the protocol-supplied surface-loss ratio must infer which specialization is reliable from model outputs. Landmark visibility alone is not necessarily equivalent to coordinate reliability, because a target can remain close to the retained surface while the supporting feature distribution has changed. The present framework therefore uses a scene-level reliability gate derived only from model outputs, followed by coordinate-consistency-constrained residual fusion. Evaluation is expanded beyond spherical removal to structured and sensor-inspired synthetic corruptions, while all claims remain restricted to controlled FaceScape conditions because no real structured-light, RGB-D, or depth-camera data were acquired.

The contributions are: (1) a dual-expert formulation separating complete-surface peak refinement from incomplete-surface structural inference; (2) an Occlusion expert that integrates local prediction, visibility, heteroscedastic modeling, and a global structural prior without explicit surface completion; (3) automatic reliability-gated residual fusion that does not use a protocol-supplied surface-loss ratio during inference; and (4) a controlled evaluation covering overall and landmark-specific localization, visible/missing subsets, parameter sensitivity, stronger routing baselines, structured corruption, computational cost, point density, paired statistics, and rigid-initialization convergence. The individual ingredients, including visibility estimation, heteroscedastic modeling, and structural priors, are established techniques; the methodological contribution lies in their specialization into independently trained complete- and incomplete-surface experts and their output-based fusion that does not require the protocol-supplied surface-loss ratio during inference for sparse rigid initialization.

## 2. Materials and Methods

### 2.1. Task Definition and Rigid Initialization Objective

Let the reference and moving facial surfaces be

(1) $$P^{\mathrm{ref}}={\left\{p_{i}^{\mathrm{ref}}\right\}}_{i=1}^{N_{\mathrm{ref}}},\;P^{\mathrm{mov}}={\left\{p_{i}^{\mathrm{mov}}\right\}}_{i=1}^{N_{\mathrm{mov}}}.$$

The network predicts five moving-surface landmarks in a fixed order: right outer canthus, left outer canthus, nasal tip, right lower nasolabial fold point, and left lower nasolabial fold point. The predicted set is ${\hat{L}}^{\mathrm{mov}}={\left\{{\hat{l}}_{k}^{\mathrm{mov}}\right\}}_{k=1}^{5}$. Given fixed reference landmarks $L^{\mathrm{ref}}={\left\{l_{k}^{\mathrm{ref}}\right\}}_{k=1}^{5}$, the coarse rigid transformation is estimated by

(2) $$\left(R^{⋆},t^{⋆}\right)=\underset{R\in SO\left(3\right),\;t\in R^{3}}{\arg\min}\sum_{k=1}^{5}{∥R{\hat{l}}_{k}^{\mathrm{mov}}+t-l_{k}^{\mathrm{ref}}∥}_{2}^{2}.$$

The singular value decomposition (SVD)-based Procrustes solution [31] yields the rigid transformation matrix $T^{⋆}=\left[R^{⋆}|t^{⋆}\right]$, which initializes optional ICP refinement [32]. The five-point configuration is evaluated only as a sparse correspondence set for rigid initialization; it is not claimed to provide a general dense facial landmark model.

### 2.2. Dataset, Splits, and Controlled Corruption Protocols

Experiments used FaceScape [21] with a subject-independent 8:1:1 split containing 13,520 training, 1690 validation, and 1690 test samples. All compared methods used the same splits, landmark annotations, and metric-space denormalization. Each point cloud was resampled to 16,384 points for the principal experiments.

The primary test conditions were 0%, 30%, and 50% controlled local surface loss. Spherical local loss was generated by selecting a surface center and removing neighboring points until the target ratio was reached. A landmark was labeled visible when its nearest distance to the remaining point cloud was below 0.09 normalized units (three heatmap widths); this label was used for training supervision and stratified analysis, but was not supplied to the final external fusion procedure.

To test whether performance was specific to spherical removal, twelve additional controlled patterns were evaluated at 30% and 50% intensity: global random reduction, contiguous local loss, left- and right-side truncation, horizontal and vertical stripes, non-uniform depth removal, targeted loss around the nasal tip and each outer canthus, local loss with coordinate quantization, and a mixed sensor-inspired perturbation. These conditions model selected missing-geometry and sampling effects but do not reproduce the full physics of any real sensor.

### 2.3. Point-Wise Representation and Dual-Expert Networks

**Figure 1:**
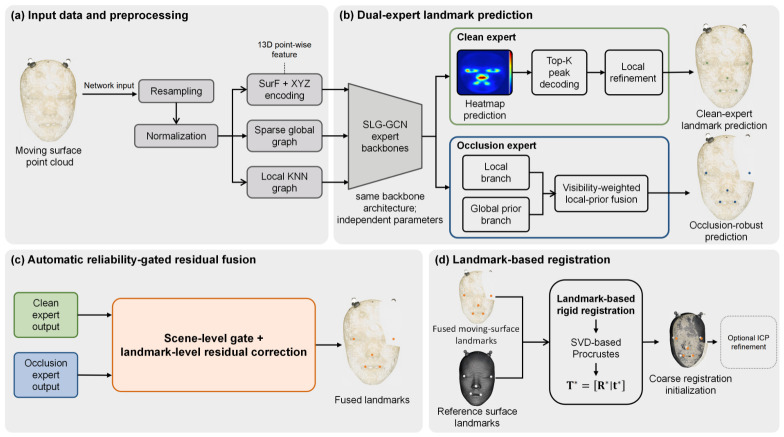
Overview of the proposed dual-expert framework for five-landmark localization and rigid initialization. The resampled and normalized moving-surface point cloud is encoded using the 13-dimensional surface feature descriptor plus Cartesian coordinates (SurF + XYZ) representation together with local *k*-nearest-neighbor (KNN) and sparse-global graph structures. The Clean and Occlusion experts use the same sparse local–global graph convolutional network (SLG-GCN) backbone architecture but have independent parameters and training objectives. Their predictions are combined through a scene-level reliability gate and landmark-level residual correction. Green, blue, orange, and white dots denote Clean-expert predictions, Occlusion-expert predictions, fused moving-surface landmarks, and reference-surface landmarks, respectively. The resulting five landmark correspondences initialize singular value decomposition (SVD)-based rigid registration, followed by optional iterative closest point (ICP) refinement.

The overall framework is shown in Figure 1. For notational simplicity, let $P={\left\{p_{i}\right\}}_{i=1}^{N}$ denote the normalized moving-surface point cloud supplied to both experts. Each normalized point is represented by its XYZ coordinate and a 10-dimensional surface feature (SurF) descriptor derived from K=8 ordered local triangular patches, producing a 13-dimensional feature vector. Local *k*-nearest-neighbor (KNN) and sparse global graphs are processed by a six-layer sparse local–global graph convolutional network (SLG-GCN) with 96 hidden channels. Detailed SurF construction and numerical safeguards are provided in Supplementary Section S1.

The Clean expert is optimized on complete surfaces and uses a peak-based coarse-to-fine decoder. For landmark *k*, let $h_{ik}^{\mathrm{clean}}$ be the Clean-expert heatmap response at point *i*, let $f_{i}^{\mathrm{clean}}$ be the corresponding point feature, and let N(i) denote the neighbors of point *i* in the local KNN graph. The graph-local heatmap-peak index set is

(3) $$P_{k}^{\mathrm{clean}}=\left\{i:h_{ik}^{\mathrm{clean}}\geq h_{jk}^{\mathrm{clean}},\;\forall j\in N\left(i\right)\right\}.$$

The $K_{p}=8$ peak indices with the largest heatmap responses are retained:

(4) $$T_{k}^{\mathrm{clean}}=\underset{i\in P_{k}^{\mathrm{clean}}}{{arg top}_{K_{p}}}\;h_{ik}^{\mathrm{clean}}.$$

Thus, candidate selection is based on heatmap response rather than Euclidean proximity. For the retained peak indices $i_{j}\in T_{k}^{\mathrm{clean}}$, the temperature-normalized decoding weights are

(5) $$\beta_{kj}=\frac{\exp\;\left(h_{i_{j}k}^{\mathrm{clean}}/T_{p}\right)}{\sum_{r=1}^{K_{p}}\exp\;\left(h_{i_{r}k}^{\mathrm{clean}}/T_{p}\right)},\;T_{p}=0.05,$$

and the peak-decoded coarse coordinate is

(6) $$c_{k}^{\mathrm{clean}}=\sum_{j=1}^{K_{p}}\beta_{kj}p_{i_{j}}.$$

A separate local feature-pooling stage selects the $K_{t}=32$ points nearest to $c_{k}^{\mathrm{clean}}$ in Euclidean coordinate space. Define the corresponding index set and distances by

$$N_{k}^{\left(K_{t}\right)}={\mathrm{KNN}}_{K_{t}}\;\left(c_{k}^{\mathrm{clean}},P\right),\;r_{kn}={∥p_{n}-c_{k}^{\mathrm{clean}}∥}_{2},\;n\in N_{k}^{\left(K_{t}\right)}.$$

The distance-softmax weights are

(7) $$\gamma_{kn}=\frac{\exp(-r_{kn}/T_{t})}{\sum_{m\in N_{k}^{\left(K_{t}\right)}}\exp\left(-r_{km}/T_{t}\right)},\;T_{t}=0.10,\;n\in N_{k}^{\left(K_{t}\right)},$$

and the pooled local feature token is

$$u_{k}^{\mathrm{clean}}=\sum_{n\in N_{k}^{\left(K_{t}\right)}}\gamma_{kn}f_{n}^{\mathrm{clean}}.$$

The pooled local token, the global mean feature token, and $c_{k}^{\mathrm{clean}}$ are concatenated and passed through a refinement multilayer perceptron (MLP), which predicts $\Delta l_{k}^{\mathrm{clean}}$. The final Clean-expert coordinate is

(8) $${\hat{l}}_{k}^{\mathrm{clean}}=c_{k}^{\mathrm{clean}}+\Delta l_{k}^{\mathrm{clean}}.$$

To avoid ambiguity, $c_{k}^{\mathrm{clean}}$ and ${\hat{l}}_{k}^{\mathrm{clean}}$ are termed the peak-decoded coarse coordinate and refined peak-based coordinate, respectively, rather than by the implementation-specific label “hard coordinate”.

The Occlusion expert contains a local coordinate branch and a global structural-prior branch. Its principal coordinate output does not use the Clean expert’s Top-*K* peak decoder. Let $h_{ik}^{\mathrm{occ}}$ and $f_{i}^{\mathrm{occ}}$ denote the Occlusion-expert heatmap response and point feature, respectively. An all-point heatmap softmax with temperature $T_{o}=1.0$ gives

(9) $$\omega_{ik}^{\mathrm{occ}}=\frac{\exp\;\left(h_{ik}^{\mathrm{occ}}/T_{o}\right)}{\sum_{n}\exp\;\left(h_{nk}^{\mathrm{occ}}/T_{o}\right)},\;c_{k}^{\mathrm{soft}}=\sum_{i}\omega_{ik}^{\mathrm{occ}}p_{i},\;z_{k}^{\mathrm{occ}}=\sum_{i}\omega_{ik}^{\mathrm{occ}}f_{i}^{\mathrm{occ}},\;T_{o}=1.0.$$

Here, $c_{k}^{\mathrm{soft}}$ is a soft coordinate and $z_{k}^{\mathrm{occ}}$ is a landmark-specific feature token. The landmark token is combined with a global face token by a context MLP, and a coordinate-regression MLP predicts a local offset $\Delta l_{k}^{\mathrm{loc}}$:

(10) $${\hat{l}}_{k}^{\mathrm{loc}}=c_{k}^{\mathrm{soft}}+\Delta l_{k}^{\mathrm{loc}}.$$

The global-prior branch independently predicts ${\hat{l}}_{k}^{\mathrm{prior}}$ from the global face token and a learned landmark-identity embedding. Let $a_{k}$ be the visibility logit. The raw visibility probability and internally clipped local-branch weight are

$$v_{k}=\mathrm{sigmoid}\left(a_{k}\right),\;{\tilde{v}}_{k}=\max\left(v_{k},0.05\right).$$

The Occlusion-expert coordinate is

(11) $${\hat{l}}_{k}^{\mathrm{occ}}={\tilde{v}}_{k}{\hat{l}}_{k}^{\mathrm{loc}}+\left(1-{\tilde{v}}_{k}\right){\hat{l}}_{k}^{\mathrm{prior}}.$$

The uncertainty head predicts an unconstrained heteroscedastic log-variance proxy $s_{k}$ following [33]. For training, the clipped value, heteroscedastic coordinate term, and raw-output scale proxy are

$${\bar{s}}_{k}=\mathrm{clip}\left(s_{k},-6,2\right),\;\ell_{k}^{\mathrm{het}}=\exp\left(-{\bar{s}}_{k}\right)\;\ell_{\mathrm{SmoothL}1,k}+{\bar{s}}_{k},\;\sigma_{k}=\exp\left(s_{k}/2\right).$$

**Figure 2:**
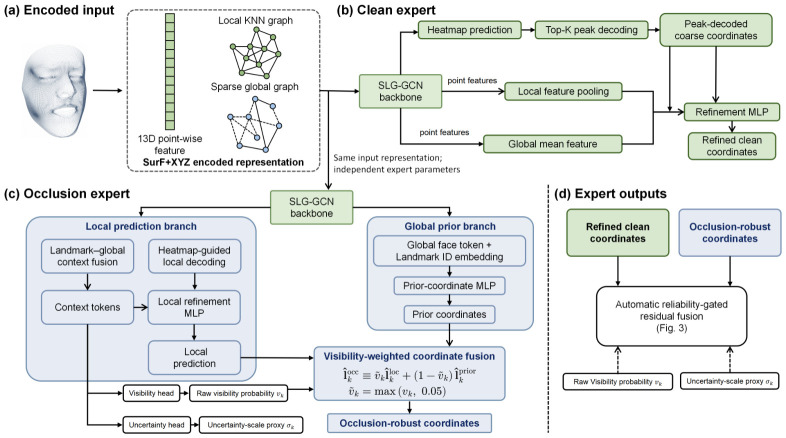
Detailed architectures of the Clean and Occlusion experts. The Clean expert performs graph-local Top-*K* peak selection and temperature-softmax coarse-coordinate decoding, followed by coarse-coordinate-centered pooling of the 32 nearest point features. The pooled local token, global mean feature, and peak-decoded coarse coordinate are jointly supplied to the refinement MLP. The Occlusion expert forms an all-point soft coordinate, predicts local and global-prior coordinates, and combines them through clipped visibility-weighted coordinate fusion. The raw visibility probability $v_{k}$ and uncertainty-scale proxy $\sigma_{k}$ are additionally supplied to the automatic scene gate in Figure 3. Visibility therefore contributes through clipped internal fusion and scene-level gating rather than serving as a stand-alone external hard-routing signal.

The scale proxy $\sigma_{k}$ is used for reliability analysis and scene gating and is not interpreted as a calibrated metric-space error estimate. Training uses heatmap supervision, coordinate regression, extra weighting for protocol-missing landmarks, balanced visibility supervision, prior-coordinate supervision on protocol-missing landmarks, and structural consistency over landmark pairs involving at least one missing landmark. During Siamese training, an auxiliary weighted Procrustes coupling loss (weight 0.2) is also applied; its landmark weights combine paired raw visibility probabilities and clipped precision-like factors $\exp(-{\bar{s}}_{k})$. The two expert architectures are summarized in Figure 2; complete objective composition, masking definitions, and weights are given in Supplementary Section S2.

### 2.4. Automatic Reliability-Gated Conservative Residual Fusion

**Figure 3:**
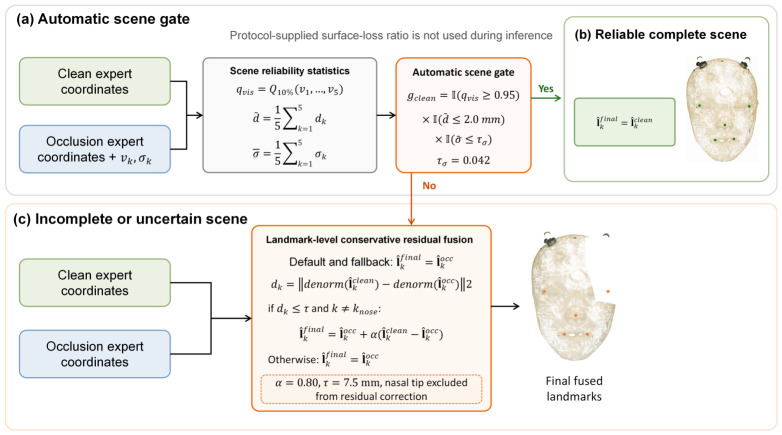
Automatic scene reliability gating and landmark-level conservative residual fusion. The gate uses only model-output statistics, including the lower-tail visibility statistic $q_{\mathrm{vis}}$, mean inter-expert discrepancy $\bar{d}$, and mean uncertainty-scale proxy $\bar{\sigma }$; the protocol-supplied surface-loss ratio is not used during inference. Reliably complete scenes use the Clean-expert coordinates directly. Otherwise, the Occlusion-expert coordinates provide the default and fallback prediction, and a validation-selected Clean residual with α=0.80 is applied only to landmarks other than the nasal tip whose inter-expert discrepancy satisfies $d_{k}\leq \tau$, where τ=7.5 mm. Green dots indicate the Clean-expert landmarks used for reliably complete scenes, whereas orange dots indicate the final fused landmarks for incomplete or uncertain scenes.

The final inference is fully determined by model outputs (Figure 3). The raw, unclipped probability $v_{k}$, rather than the internally clipped weight ${\tilde{v}}_{k}$, is used for visibility calibration and scene-level reliability assessment. For a scene with five landmarks, define the lower-tail visibility statistic, mean inter-expert discrepancy, and mean scale proxy as

(12) $$q_{\mathrm{vis}}=Q_{10\%}\left(v_{1},\ldots ,v_{5}\right),\;\bar{d}=\frac{1}{5}\sum_{k=1}^{5}d_{k},\;\bar{\sigma }=\frac{1}{5}\sum_{k=1}^{5}\sigma_{k}.$$

The statistic $q_{\mathrm{vis}}$ is the 10th percentile of the five raw visibility probabilities. It was chosen as a conservative lower-tail summary: the gate opens only when the landmark-wise visibility outputs are consistently high, while the interpolated lower-tail quantile is slightly less sensitive than the strict minimum to a single anomalous output. $Q_{10\%}$ is computed using the default linear-interpolation quantile definition in NumPy (v1.26.4) and PyTorch (v2.1.0). The landmark-wise inter-expert discrepancy in metric space is

(13) $$d_{k}={∥\mathrm{denorm}\;\left({\hat{l}}_{k}^{\mathrm{clean}}\right)-\mathrm{denorm}\;\left({\hat{l}}_{k}^{\mathrm{occ}}\right)∥}_{2}.$$

Here, I(·) denotes the indicator function. The scene-level Clean-expert reliability indicator is

(14) $$g_{\mathrm{clean}}=I\;\left(q_{\mathrm{vis}}\geq 0.95\right)I\;\left(\bar{d}\leq 2.0\;\mathrm{mm}\right)I\;\left(\bar{\sigma }\leq \tau_{\sigma }\right).$$

The gate thresholds were selected using validation outputs only. The visibility cutoff was searched over {0.40,0.50,0.60,0.70,0.80,0.90,0.95}, the mean-discrepancy cutoff over {0.5,1,1.5,2,3,4,5,7.5,10} mm, and the uncertainty cutoff over the 0.50, 0.60, 0.70, 0.80, 0.90, and 0.95 validation quantiles together with the validation maximum. Candidate gates were ranked by validation sample-level mean error, then by the failure rate above 6 mm, and finally by the Clean-gate rate. This procedure selected $q_{\mathrm{vis}}\geq 0.95$, $\bar{d}\leq 2.0$ mm, and $\tau_{\sigma }=0.042$ (implementation value 0.0420783). Thus, 0.042 is a validation-selected operating threshold rather than a theoretically universal constant. For scenes that do not satisfy the Clean-expert gate, define the validation-selected residual parameters

$$\alpha =0.80,\;\tau =7.5\;\mathrm{mm},\;k_{\mathrm{nose}}=3,$$

where $k_{\mathrm{nose}}=3$ denotes the nasal tip under the one-based landmark ordering of Section 2.1 (zero-based implementation index 2). The landmark-wise residual-activation mask is

(15) $$m_{k}=I\;\left(d_{k}\leq \tau \right)I\;\left(k\neq k_{\mathrm{nose}}\right).$$

The complete final inference rule is

(16) $${\hat{l}}_{k}^{\mathrm{final}}=\left\{\begin{array}{cc} {\hat{l}}_{k}^{\mathrm{clean}},\; & g_{\mathrm{clean}}=1,\\ {\hat{l}}_{k}^{\mathrm{occ}}+\alpha m_{k}\left({\hat{l}}_{k}^{\mathrm{clean}}-{\hat{l}}_{k}^{\mathrm{occ}}\right),\; & g_{\mathrm{clean}}=0. \end{array}\right.$$

The discrepancies $d_{k}$, the mean discrepancy $\bar{d}$, and all distance thresholds are evaluated after metric-space denormalization. The coordinate interpolation in Equation (16) is performed in the normalized coordinate space, and the fused coordinates are subsequently denormalized for evaluation and registration. The residual search evaluated α∈{0,0.2,0.4,0.6,0.8,1.0}, discrepancy thresholds of {1,2,3,4,5,7.5,10} mm, and either no landmark exclusion or exclusion of each individual landmark. Candidate configurations were ranked using occluded validation scenes by sample-level mean error, then by the failure rate above 6 mm, followed by deterministic tie breakers. Test-set performance was not used for selection. The selected configuration (α=0.80, τ=7.5 mm, nasal-tip exclusion) was recovered in all 200 bootstrap resamples of the validation set. The protocol-supplied surface-loss ratio was used only to generate and stratify controlled test conditions and was not an input to Equations (12)–(16).

### 2.5. Comparators, Statistics, and Computational Analysis

Comparators included the two-stage stratified graph convolutional network (2S-SGCN) heatmap baseline, a PointNet++ baseline, the Unified occlusion-aware and structure-robust (OASR) model, the two individual experts, simple averaging, predicted-visibility hard routing, uncertainty-weighted fusion, an uncertainty-only learned router, learned soft and hard mixture-of-experts (MoE) routers, a learned residual gate, and a ground-truth-dependent best-expert oracle. Unified OASR served as the jointly trained shared-backbone, multi-output-head single-network comparator: one SLG-GCN backbone generated the common heatmap and point-feature representation used by its coordinate-regression, visibility, and heteroscedastic uncertainty heads. Its stable pred_coords output was used consistently in every comparison.

Learned routers were fitted on validation predictions and evaluated once on the independent test set, so they compare fusion strategies without retraining the experts.

Localization error was the 3D Euclidean distance in millimeters. Results include mean ± standard deviation (SD), median, failure rate above 6 mm, visibility-stratified errors, and landmark-wise errors. Paired sample-level comparisons used two-sided Wilcoxon signed-rank tests. Holm’s sequentially rejective procedure controlled the family-wise error rate at α=0.05 within each prespecified comparison family; the four primary localization comparisons and the twelve comparator–metric registration comparisons were corrected separately [34]. Reported values are Holm-adjusted *p* values. Paired bootstrap confidence intervals were calculated for mean improvement. Visibility calibration was assessed using accuracy, balanced accuracy, area under the receiver operating characteristic curve (AUROC), area under the precision–recall curve (AUPRC), Brier score, and expected calibration error (ECE). Inference latency, parameter count, checkpoint size, and peak graphics processing unit (GPU) memory were measured on 16,384-point inputs using an NVIDIA GeForce RTX 4090D GPU (NVIDIA Corporation, Santa Clara, CA, USA) with CUDA 12.1 and sequential expert execution.

### 2.6. Controlled Large-Pose Registration Stress Test

A separate stress test evaluated whether landmark initialization changed ICP convergence under a deliberately difficult pose. Surface root mean square error (RMSE) was recorded before and after ICP. Each 30%-loss test surface was flipped by 180° around the *x* axis, translated by approximately (80,80,120) mm, and perturbed by up to ±10 mm, with five trials per sample (8450 trials). ICP success required rotation error below 3° and translation error below 3 mm. The dual-expert coordinates were generated using the same final automatic reliability gate, α=0.80, 7.5 mm discrepancy threshold, and nasal-tip residual exclusion as in Section 2.4. The 30% loss ratio was used only to generate the controlled moving surfaces and was not supplied to the fusion procedure. The archived perturbation transforms were reused exactly across initialization methods. Descriptive registration results are reported over all trials; paired inference comparisons use one trial-averaged value per test sample to avoid treating repeated trials as independent observations.

## 3. Results

### 3.1. Primary Localization Performance and Paired Comparisons

Table 1 reports the primary test results. The automatic method retained near-Clean performance at 0% loss and reduced error relative to Unified OASR and the Occlusion expert at 30% and 50%. At 30%, it improved the sample-level mean by 0.658 mm relative to Unified OASR (95% bootstrap confidence interval (CI), 0.618–0.698 mm) and by 0.236 mm relative to the Occlusion expert (0.224–0.247 mm). At 50%, the corresponding improvements were 2.409 mm (2.271–2.550 mm) and 0.452 mm (0.437–0.468 mm). All four Holm-adjusted *p* values were below 0.001; 0.001 was the reporting level of the adjusted results, not the prespecified significance threshold.

**Figure 4:**
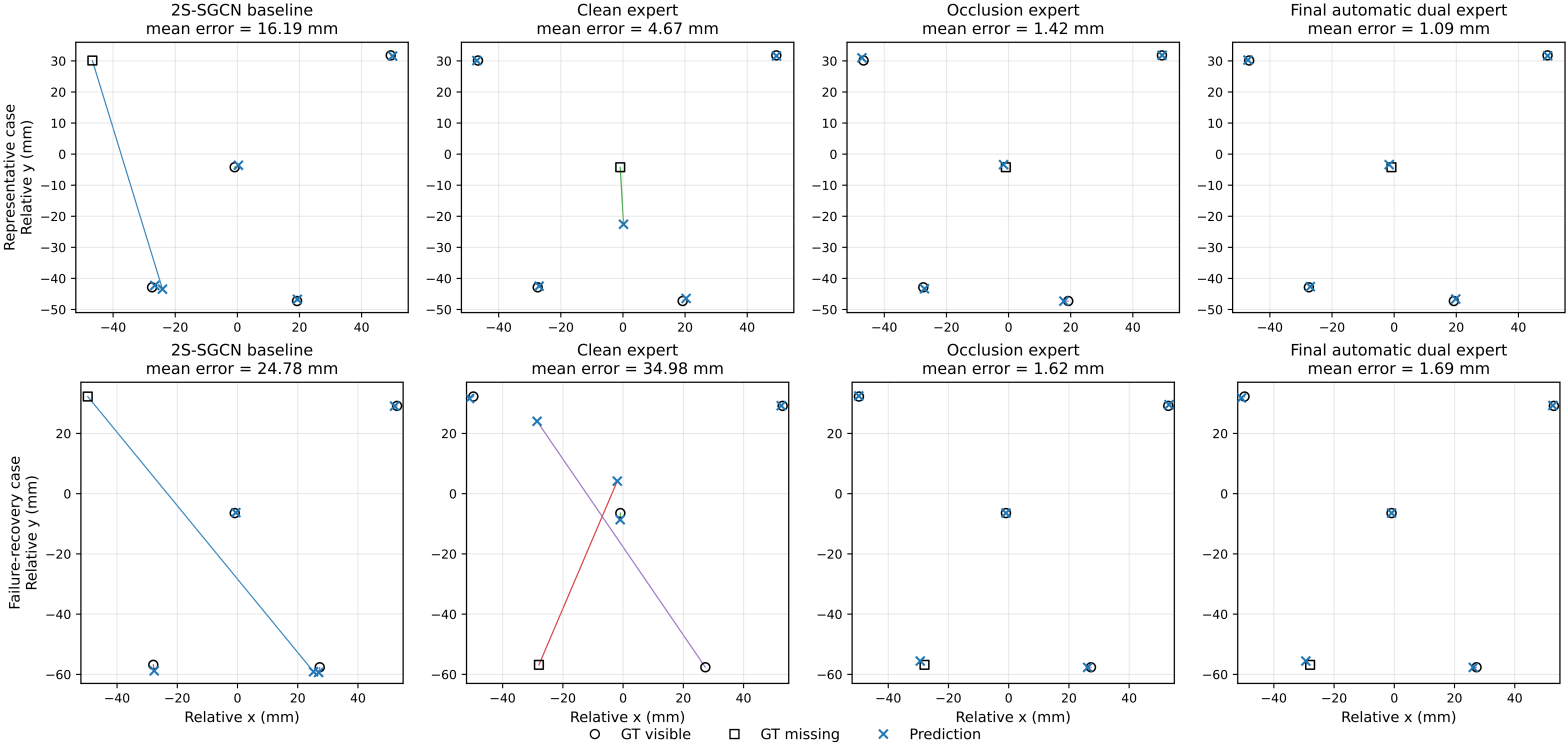
Representative and failure-recovery cases at 30% controlled local surface loss. Landmark positions are shown in the *x*–*y* projection, whereas panel titles report the sample-level mean 3D Euclidean error. Circles denote protocol-visible ground-truth landmarks, squares denote ground-truth landmarks in protocol-defined missing regions, crosses denote predictions, and connecting-line colors distinguish landmark identities while showing projected ground-truth-to-prediction displacement.

**Figure 5:**
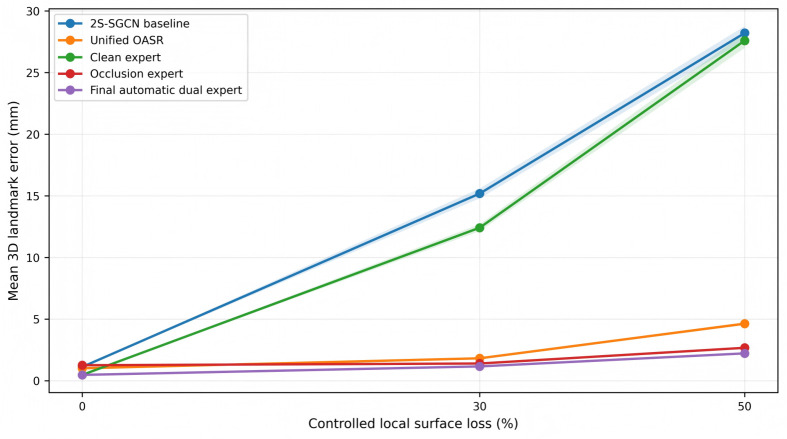
Mean 3D landmark error at 0%, 30%, and 50% controlled local surface loss. Shaded regions are sample-level bootstrap 95% confidence intervals.

Representative 30% cases are shown in Figure 4. The first row illustrates a typical sample; the second shows recovery from large baseline and Clean-expert displacements. Figure 5 summarizes the three primary loss levels with sample-level bootstrap confidence bands.

### 3.2. Automatic Scene Selection and Validation-Only Fusion Design

The final scene gate selected the Clean branch for 98.22% of complete surfaces, 3.96% of 30%-loss surfaces, and none of the 50%-loss surfaces (Table 2). Across the equally sized groups, agreement with the protocol-defined complete/non-complete grouping was 98.09%. Replacing the protocol-stratified scene switch with automatic gating changed the validation-selected residual results only from 0.467/1.146/2.216 mm to 0.470/1.159/2.216 mm at 0%/30%/50%, respectively.

**Figure 6:**
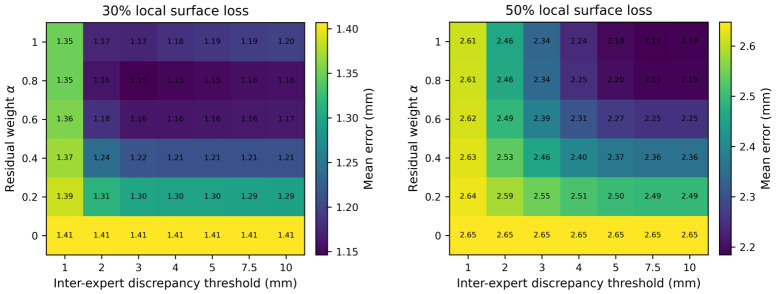
Validation sensitivity of residual weight and inter-expert discrepancy threshold at 30% and 50% controlled loss with nasal-tip residual exclusion. The test set was not used for parameter selection.

The selected design excluded the nasal tip from Clean residual correction. Nasal-tip exclusion was selected using validation data and is therefore treated as a dataset- and landmark-configuration-specific safeguard ($k_{\mathrm{nose}}=3$ under the one-based mathematical ordering; zero-based implementation index 2), rather than as a universal anatomical rule. Figure 6 shows the validation surfaces for residual weight and discrepancy threshold. The selected combination was recovered in all 200 bootstrap repetitions, demonstrating stability across validation resamples.

### 3.3. Stronger Fusion Baselines and Reliability Outputs

Table 3 shows that neither simple averaging nor visibility-only routing reproduced the performance of the final rule. Predicted-visibility hard routing produced 3.080 and 8.031 mm at 30% and 50% loss, even though visibility prediction itself was accurate. Learned MoE routers were competitive, but the deterministic final rule achieved the lowest aggregate error among inference-time applicable strategies and required no additional routing model.

The visibility output showed high classification performance and low aggregate calibration error for the controlled visibility labels, whereas the uncertainty-scale proxy was not a calibrated estimate of coordinate error (Table 4). This weak association is consistent with the poor performance of uncertainty-weighted fusion. In the final gate, uncertainty is used only as a secondary scene-level veto together with lower-tail visibility and inter-expert disagreement.

### 3.4. Landmark-Wise and Visibility-Stratified Performance

Table 5 reports each anatomical point and separates protocol-visible from protocol-missing subsets. At 50% loss, the nasal tip and bilateral lower nasolabial fold points remained the most difficult. The aggregate missing-landmark error increased from 2.317 mm at 30% to 3.896 mm at 50%, whereas visible-landmark error increased from 0.931 to 1.462 mm.

### 3.5. Structured Corruption and Training-Design Analyses

**Figure 7:**
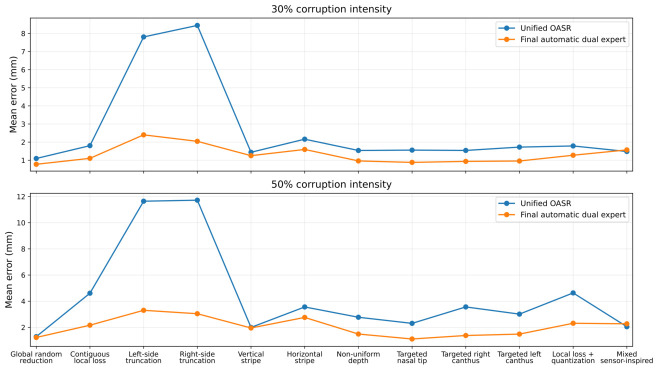
Pattern-wise comparison under twelve controlled structured and sensor-inspired synthetic corruptions. These experiments evaluate controlled synthetic perturbations and do not constitute validation using data acquired from real sensors.

Across twelve structured patterns, the final method averaged 1.313 mm at 30% and 2.042 mm at 50% intensity, compared with 2.698 and 4.431 mm for Unified OASR (Table 6; Figure 7). It was better in 11 of 12 patterns at each intensity. Unified OASR was slightly better for the mixed sensor-inspired perturbation at both intensities. The result therefore supports robustness to several controlled missing-geometry patterns, not general real-sensor robustness.

The training ablations in Table 7 show that removing occlusion-specific training caused the largest degradation in the evaluated ablation series and that removal of the global prior, occluded-coordinate emphasis, or structural consistency increased error. Removing visibility supervision did not consistently increase coordinate error, but it reduced visibility accuracy to 16.86%, limiting the output’s utility for automatic scene gating.

### 3.6. Computational Cost and Point-Density Sensitivity

The dual system increased parameter count and latency relative to a single network (Table 8). Relative to Unified OASR, it used approximately 2.15 times as many parameters and 39.1% more mean inference time; sequential execution limited the peak-memory increase to approximately 2.5 MB. Point-density sensitivity was evaluated at 4096, 8192, and 16,384 points using the complete final automatic inference procedure at every density (Table 9). The largest difference in mean error was approximately 0.001 mm across the tested densities, but this controlled result should not be extrapolated to arbitrary real-sensor sparsity or non-uniform sampling.

### 3.7. Controlled Large-Pose Rigid-Initialization Stress Test

**Figure 8:**
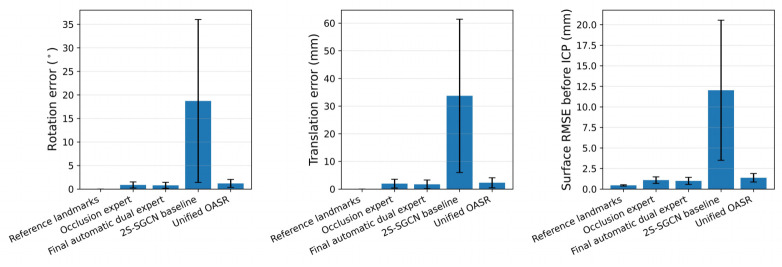
Coarse-initialization metrics in the large-pose stress test. Bars and error bars show mean ± SD over 8450 trials. Final ICP errors are reported separately because the successful landmark-based initializers converged to essentially the same solution under this controlled test.

Direct ICP succeeded in only 0.05% of the deliberately difficult trials. Landmark initialization increased success to 98.82% for the heatmap baseline and 100% for the Occlusion expert, Unified OASR, and the final automatic dual-expert method (Table 10). Relative to Unified OASR, the final dual-expert coordinates reduced sample-level coarse rotation error by 0.407° (95% bootstrap CI, 0.373–0.441°), coarse translation error by 0.575 mm (0.506–0.647 mm), and pre-ICP surface RMSE by 0.381 mm (0.359–0.403 mm). The corresponding Holm-adjusted *p* values were below 0.001, and the dual method was better for 73.49%, 65.62%, and 82.72% of test subjects, respectively. It also improved all three coarse metrics relative to the Occlusion expert. By contrast, post-ICP rotation, translation, and surface RMSE did not differ significantly from Unified OASR or the Occlusion expert after Holm correction. These results support improved coarse initialization and convergence, not improved final ICP accuracy after successful convergence. The three coarse-initialization metrics are summarized in Figure 8, and the corresponding sample-level paired comparisons are reported in Table 11.

## 4. Discussion

The results support the value of specialization across complete and locally incomplete surfaces. The Clean expert achieved the best complete-surface precision but exhibited large outliers when target neighborhoods were removed. The Occlusion expert traded complete-surface precision for substantially lower missing-region error by combining local evidence with a global structural prior. The automatic scene gate preserved this specialization without receiving the protocol-supplied surface-loss ratio.

Visibility and coordinate reliability were related but not interchangeable. Although the visibility head was highly accurate for the controlled labels, visibility hard routing remained poor. A landmark can remain protocol-visible while the Clean prediction becomes unreliable because contextual geometry and the network feature distribution have changed. Likewise, the weak association between the predicted uncertainty-scale proxy and coordinate error is consistent with the poor performance of uncertainty-weighted fusion. The final rule therefore combines scene-level visibility, disagreement, and uncertainty, while landmark-level residual activation is determined by coordinate agreement.

The final parameter configuration was selected exclusively from validation-set outputs; test-set performance was not used to rank the candidate configurations. Specifically, the selected residual weight of 0.80, the 7.5 mm inter-expert discrepancy threshold, and nasal-tip exclusion were recovered in all 200 validation bootstrap resamples. Nevertheless, these values remain empirical operating parameters for the present dataset and five-landmark configuration. Similarly, the point-density analysis demonstrates stability only over the tested range of 4096 to 16,384 points under controlled FaceScape resampling and should not be extrapolated to arbitrary device-specific sparsity or non-uniform sampling.

The structured-corruption results broaden the evidence beyond spherical removal and show strong gains for contiguous, side-specific, stripe, depth-dependent, targeted, and quantized missing geometry. The smaller or reversed gain under mixed sensor-inspired perturbation defines an important boundary: dual-expert specialization is most useful when the dominant failure is localized or structured surface loss. These synthetic transformations do not reproduce real structured-light shadows, reflective failures, motion, accessories, device-specific depth noise, or cross-domain geometry in their full complexity.

Training ablations show that the method’s robustness depends on both data exposure and structural modeling. Occlusion-specific training was critical under the evaluated setting. The global prior, missing-coordinate emphasis, and structural consistency each reduced error. Visibility supervision primarily produced the reliability signal required by the automatic gate; its removal did not monotonically worsen coordinate error, indicating that its principal role was reliability estimation rather than an independent and consistently positive contribution to coordinate accuracy.

The registration analysis confirms a narrower downstream role. Under large initial displacement, direct ICP almost always failed, whereas accurate landmark initializers converged reliably. The stress test used the same final automatic inference procedure as the primary localization analysis, ensuring consistency between the localization and registration evaluations. The dual-expert coordinates improved the coarse transform relative to both Unified OASR and the Occlusion expert, but no significant difference in final ICP accuracy was observed after convergence. The method is therefore a coarse-initialization front end rather than a replacement for dense registration.

Beyond rigid registration, the predicted sparse landmarks may also serve as geometric anchors in geometry-aware avatar-generation and animation pipelines. When facial regions are incomplete, occlusion-robust landmarks could support facial canonicalization, rigid pose normalization, cross-view registration, and stabilization of depth- or geometry-conditioned generation. This perspective is complementary to recent geometry-recovery systems such as Depth Anything 3, which estimates spatially consistent geometry from one or more visual inputs, and multimodal avatar-generation frameworks such as MAVAGEN, which explicitly incorporate landmark-based pose and depth-geometry features [35,36]. In such pipelines, the proposed method would provide an initialization or consistency cue rather than replace dense geometry recovery or generative modeling. This downstream use was not evaluated in the present study and therefore requires future validation on real sensor-derived facial point clouds and on depth maps or point clouds reconstructed from images.

The principal limitations are the single FaceScape domain, synthetic corruption, five-landmark task, and increased latency from two independent networks. Real structured-light, RGB-D, depth-camera, and sensor-to-reference experiments are required before making deployment or clinical claims. The five landmarks are evaluated because they form a sparse rigid-correspondence set; the study does not establish general dense or high-cardinality facial landmark detection.

## 5. Conclusions

A dual-expert model with automatic scene reliability gating improved five-landmark localization under controlled synthetic local surface loss on FaceScape. The final localization procedure no longer uses a protocol-supplied surface-loss ratio, and validation-selected residual fusion was competitive with learned routers while remaining deterministic. Landmark-wise, structured-corruption, calibration, computational, density, and ablation analyses clarify both the sources and limits of the observed gains. A large-pose stress test further showed that accurate landmark coordinates can improve coarse alignment and ICP convergence, although no significant improvement in final ICP accuracy was observed once convergence was achieved. Potential use as a geometric anchor for facial canonicalization and geometry-conditioned avatar pipelines remains a future research direction rather than an established outcome. Validation on real sensors, image-derived depth or point clouds, and cross-domain facial geometry remains necessary before such use can be claimed.

## Figures and Tables

**Table 1:** Landmark localization under the primary controlled conditions. Values are calculated over 8450 landmarks per condition. Unified OASR is evaluated using its stable coordinate-regression output. Abbreviations: SD, standard deviation; 2S-SGCN, two-stage stratified graph convolutional network; OASR, occlusion-aware and structure-robust.

Method	0% Mean ± SD (mm)	30% Mean ± SD (mm)	30% Median (mm)	50% Mean ± SD (mm)	50% Median (mm)	50% > 6 mm (%)
2S-SGCN baseline	1.114±0.763	15.185±31.282	1.473	28.212±39.489	2.784	43.30
PointNet++ baseline	0.665±0.606	12.448±26.157	0.677	27.276±35.049	8.588	43.48
Unified OASR	1.006±0.683	1.816±1.858	1.276	4.626±4.209	3.424	23.74
Clean expert	0.467±0.649	12.414±23.902	1.089	27.598±34.075	2.139	45.09
Occlusion expert	1.254±0.710	1.394±0.979	1.203	2.669±1.982	2.119	6.56
Final automatic dual expert	0.470±0.688	1.159±1.093	0.858	2.216±2.169	1.437	6.63

**Table 2:** Automatic gate behavior and residual-parameter justification. The aggregate is the equally weighted mean across 0%, 30%, and 50% test groups. Aggregate values were calculated from unrounded condition-level means and may therefore differ by 0.001 mm from arithmetic based on the displayed rounded values.

Inference/Design Setting	0% (mm)	30% (mm)	50% (mm)	Aggregate (mm)	Additional Evidence
Original protocol-stratified rule, α=0.40, τ=3 mm	0.467	1.205	2.473	1.381	Original setting
Validation-selected protocol-stratified rule, α=0.80, τ=7.5 mm	0.467	1.146	2.216	1.276	200/200 bootstrap selections
Final automatic gate + selected residual	0.470	1.159	2.216	1.281	Gate rates: 98.22%, 3.96%, and 0.00%

**Table 3:** Fusion-strategy comparison. “All” is the aggregate mean over equally sized 0%, 30%, and 50% test groups. Aggregate values were calculated from unrounded condition-level means and may therefore differ by 0.001 mm from arithmetic based on the displayed rounded values.

Strategy	0% (mm)	30% (mm)	50% (mm)	All (mm)
Unified OASR	1.006	1.816	4.626	2.483
Occlusion expert	1.254	1.394	2.669	1.772
Simple average	0.741	6.525	14.123	7.130
Visibility hard routing	0.467	3.080	8.031	3.859
Uncertainty-weighted soft fusion	0.610	6.342	9.190	5.381
Uncertainty-only learned hard router	0.611	6.740	6.837	4.729
Learned residual gate	0.528	1.405	2.777	1.570
Learned MoE soft router	0.538	1.142	2.245	1.308
Learned MoE hard router	0.515	1.161	2.222	1.299
Final automatic dual expert	0.470	1.159	2.216	1.281
Best-expert oracle	0.404	1.053	2.126	1.194

**Table 4:** Reliability diagnostics for the Occlusion expert over the three primary test groups. The association between the uncertainty-scale proxy and coordinate error is reported using Spearman correlation.

Visibility Accuracy	Balanced Accuracy	AUROC	AUPRC	Brier	ECE	Spearman ρ
98.87%	98.44%	0.99895	0.99979	0.00862	0.00486	0.092

**Table 5:** Landmark-wise and visibility-stratified performance of the final automatic method.

Landmark	30% Mean ± SD (mm)	30% Visible (mm)	30% Missing (mm)	50% Mean ± SD (mm)	50% Visible (mm)	50% Missing (mm)
Right outer canthus	1.003±1.102	0.780	2.038	1.543±1.867	1.035	3.358
Left outer canthus	0.873±0.789	0.678	1.705	1.196±1.315	0.779	2.568
Nasal tip	1.442±0.917	1.307	1.965	3.200±1.701	3.149	3.253
Right lower nasolabial fold point	1.312±1.201	1.015	3.209	2.560±2.418	1.479	5.046
Left lower nasolabial fold point	1.162±1.281	0.881	3.352	2.584±2.619	1.446	5.200
All landmarks	1.159±1.093	0.931	2.317	2.216±2.169	1.462	3.896

**Table 6:** Summary across twelve controlled structured and sensor-inspired synthetic corruption patterns.

Intensity	Final Automatic (mm)	Unified OASR (mm)	Final Wins	Final-Method Mean > 6 mm Rate
30%	1.313	2.698	11/12	1.50%
50%	2.042	4.431	11/12	6.22%

**Table 7:** Occlusion-expert training-design ablations. The full-model row is the within-ablation reference.

Variant	30% Mean ± SD (mm)	50% Mean ± SD (mm)	30% Missing (mm)	30% > 6 mm (%)	Visibility Accuracy
Full model (ablation run)	1.221±1.052	2.470±2.115	2.358	0.53	98.95%
Without occlusion-specific training	13.817±5.456	22.234±7.125	18.615	94.78	83.14%
Without visibility supervision	1.263±0.995	2.002±1.659	2.224	0.30	16.86%
Without global prior branch	1.686±1.352	2.719±3.119	2.801	1.80	98.97%
Without occluded-coordinate emphasis	1.530±1.299	2.595±1.983	2.903	1.55	98.88%
Without structural consistency	1.747±1.389	2.997±2.015	3.152	1.92	98.70%

**Table 8:** Computational cost for a 16,384-point input. Latency is mean ± SD.

Method	Parameters	Checkpoint (MB)	Mean Latency (ms)	95th-Percentile Latency (ms)	Peak GPU Memory (MB)
2S-SGCN baseline	206,309	2.14	131.3±1.1	133.0	444.0
Unified OASR	323,863	3.12	162.3±1.9	165.9	444.9
Clean expert	302,612	3.16	158.4±1.5	161.0	444.8
Occlusion expert	393,786	4.33	158.1±1.8	160.7	445.1
Dual expert (sequential)	696,398	7.49	225.7±1.6	228.2	447.4

**Table 9:** Point-density sensitivity of the final automatic inference procedure in the controlled FaceScape protocol. Density experiments were rerun independently with density-specific resampling and a fixed random seed; therefore, rounded 16,384-point values may differ from the primary evaluation by approximately 0.001 mm.

Input Points	0% (mm)	30% (mm)	50% (mm)
4096	0.469	1.160	2.217
8192	0.469	1.159	2.217
16,384	0.470	1.159	2.217

**Table 10:** Large-pose registration stress test at 30% controlled local surface loss (1690 samples, five repeated trials per sample; 8450 trials). The dual-expert row uses the same final automatic gate and validation-selected residual parameters as the primary localization evaluation.

Initialization	Coarse Rot. Error (°)	Coarse Trans. Error (mm)	Pre-ICP RMSE (mm)	ICP Success (%)	Final ICP RMSE (mm)
Direct ICP	–	–	68.364±7.051	0.05	68.807±125.940
Reference landmarks	0.000±0.000	0.000±0.000	0.446±0.076	100.00	0.446±0.076
Occlusion expert	0.876±0.641	1.949±1.593	1.084±0.400	100.00	0.446±0.076
2S-SGCN baseline	18.711±17.303	33.709±27.725	12.024±8.525	98.82	0.779±3.104
Unified OASR	1.191±0.841	2.271±1.801	1.369±0.518	100.00	0.446±0.076
Final automatic dual expert	0.784±0.642	1.696±1.564	0.988±0.430	100.00	0.446±0.076

**Table 11:** Sample-level paired comparison of the final automatic dual expert against Unified OASR in the large-pose stress test. Positive improvement favors the final method; confidence intervals are paired bootstrap 95% intervals over 1690 test samples.

Metric	Mean Improvement	95% CI	Final Better (%)	Holm *p*
Coarse rotation error	0.407°	[0.373, 0.441]	73.49	<0.001
Coarse translation error	0.575 mm	[0.506, 0.647]	65.62	<0.001
Pre-ICP surface RMSE	0.381 mm	[0.359, 0.403]	82.72	<0.001
Post-ICP rotation error	<0.001°	[−0.000005, 0.000007]	51.07	0.487
Post-ICP translation error	−0.000005 mm	[−0.000025, 0.000015]	48.82	1.000
Post-ICP surface RMSE	<0.000001 mm	[−0.000001, 0.000002]	50.71	1.000

## Data Availability

Restrictions apply to the availability of the FaceScape source data, which were obtained under the dataset’s original access terms and license. The implementation code and trained model checkpoints are not publicly available because they form part of an ongoing proprietary commercial project and are subject to company confidentiality and intellectual-property restrictions. To support methodological transparency within these constraints, the network architecture, training objectives, loss weights, validation procedures, automatic-inference rules, controlled corruption protocols, evaluation metrics, statistical procedures, and numerical results are reported in the article and Supplementary Materials. The FaceScape source data must be obtained under the dataset’s original access terms and license.

## Supplementary Materials

The following supporting information can be downloaded at https://www.mdpi.com/article/10.3390/s26154989/s1: Figure S1: Surface feature descriptor plus Cartesian coordinates (SurF + XYZ) point-wise feature construction. K-nearest neighbors (KNN; $K_{s}=8$) are ordered by azimuthal angle to form local triangular patches. CP, centroid position; N, triangle normal; SC, spherical coordinates; SP, centroid-normal projection; MLP, multilayer perceptron; Figure S2: Validation sensitivity surfaces at 30% and 50% loss with nasal-tip exclusion; Figure S3: Visibility calibration and uncertainty risk–coverage diagnostics. Visibility showed high classification performance and low aggregate calibration error for the controlled synthetic labels, whereas uncertainty was only weakly associated with localization error; Figure S4: Landmark-wise mean errors for the final automatic method at 30% and 50% loss; Figure S5: Point-density sensitivity within the controlled FaceScape protocol; Figure S6: Representative large-pose stress-test projections before coarse initialization, after landmark-based initialization, and after ICP; Figure S7: Surface root mean square error (RMSE) before and after iterative closest point (ICP) refinement in the large-pose stress test. Bars and error bars show mean ± SD over 8450 trials; Table S1: Correspondence between main-text topics and supplementary evidence; Table S2: Implementation and training settings extracted from the archived project configuration and model definitions; Table S3: Original and validation-selected residual settings on the test set; Table S4: Automatic scene-gate behavior on the independent test set; Table S5: Full primary localization summaries at 0%, 30%, and 50% controlled local surface loss. Failure rates are calculated over 8450 landmarks per condition. Unified OASR uses its stable coordinate-regression output (pred_coords); Table S6: Visibility calibration and uncertainty–error association. AUROC, area under the receiver operating characteristic curve; AUPRC, area under the precision–recall curve; ECE, expected calibration error; Table S7: Final automatic method by landmark and protocol-defined visibility. *N* visible/missing reports the subset sizes; all errors are in millimeters; Table S8: Full fusion-strategy comparison. Errors are mean millimeters; Table S9: Detailed fusion diagnostics at 30% controlled local surface loss. Mean Clean weight is the average coordinate weight assigned to the Clean expert; Table S10: Detailed fusion diagnostics at 50% controlled local surface loss; Table S11: Pattern-wise results for final automatic fusion versus Unified OASR. Difference is final minus Unified; negative values favor the final method; Table S12: Paired sample-level comparisons for the final automatic method. Positive improvement favors the final method; intervals are paired bootstrap 95% confidence intervals over 1690 test samples; Table S13: Model size, latency, and graphics processing unit (GPU) memory on 16,384-point inputs; Table S14: Final automatic inference across input point counts. Density experiments were rerun independently with density-specific resampling and a fixed random seed; rounded 16,384-point values may therefore differ from the primary evaluation by approximately 0.001 mm; Table S15: Occlusion-expert training ablations. The full-model row is the within-ablation reference; Table S16: Large-pose stress-test results using final automatic dual-expert inference; Table S17: Sample-level paired comparison of final automatic dual-expert registration outputs. Positive improvement favors the final automatic dual expert. Holm correction was applied across the twelve comparator–metric tests.

## Abbreviations

2S-SGCN	Two-stage stratified graph convolutional network
AUPRC	Area under the precision–recall curve
AUROC	Area under the receiver operating characteristic curve
CI	Confidence interval
ECE	Expected calibration error
GPU	Graphics processing unit
ICP	Iterative closest point
KNN	*k*-nearest neighbors
MLP	Multilayer perceptron
MoE	Mixture of experts
OASR	Occlusion-aware and structure-robust model
RGB-D	Red–green–blue and depth
RMSE	Root mean square error
SD	Standard deviation
SLG-GCN	Sparse local–global graph convolutional network
SVD	Singular value decomposition
SurF	Surface feature descriptor
XYZ	Cartesian coordinates
