# Supplementary material for: Dual-Expert Landmark Localization in 3D Facial Point Clouds Under Controlled Synthetic Local Surface Loss for Rigid Initialization
Source: Sensors (Basel). 2026 Aug 6;26(15):4989. doi: 10.3390/s26154989 (PMC13468751; doi:10.3390/s26154989)
Supplement: Supplementary file 1 [file sensors-26-04989-s001.zip › sensors-4460268-supplementary.pdf]

Supplementary Materials

Supplementary Methods and Extended Results

Dual-Expert Landmark Localization in 3D Facial Point Clouds under Controlled Synthetic Local Surface Loss for Rigid Initialization Sensors

Zichao Zou[1], Zongjian Chen[1], Rongqian Yang[2], Kehai Peng[3], and Shizhong Jiang[1],*

[1] College of Medical Information Engineering, Guangdong Pharmaceutical University, Guangzhou 510006, Guangdong, China.

[2] School of Electronic and Information Engineering, South China University of Technology, Guangzhou 510640, Guangdong, China.

[3] Department of Research and Development, Guangzhou Aimooe Technology Co., Ltd., Guangzhou 510535, Guangdong, China.

* Corresponding author: jiangshizhong@gdpu.edu.cn

Overview

This supplement supports the manuscript, provides implementation traceability, and distinguishes the final automatic inference without the protocol-supplied surface-loss ratio from protocol-stratified reference analyses. The final localization method uses the scene gate $q_{0.1}(v)\geq 0.95$, mean expert gap $d\leq 2.0$mm, mean uncertainty-scale proxy $\sigma\leq 0.042078$, and, for non-clean scenes, residual weight $\alpha=0.80$ with a 7.5 mm landmark-level discrepancy threshold and nasal-tip exclusion. The final parameter configuration was selected exclusively from validation-set outputs; test-set performance was not used to rank the candidate configurations. The ground-truth surface-loss ratio is used to generate and stratify controlled conditions but is not supplied to final inference.

Table S1: Correspondence between main-text topics and supplementary evidence.

Main-text topic	Supporting material
SurF+XYZ construction and normalization	Section S1; Fig. S1; Table S2
Clean peak-based decoder and Occlusion local/prior branches	Sections S2.1–S2.2; Table S2
Complete objective composition and masking	Section S2.3; Table S2
Automatic inference without the protocol-supplied surface-loss ratio	Sections S3–S4; Tables S3–S5; Fig. S2
Calibration and landmark-wise behavior	Section S5; Tables S6–S7; Figs. S3–S4
Stronger fusion baselines	Section S6; Tables S8–S10
Structured synthetic corruptions	Section S7; Table S11; main-text Fig. 7
Paired localization statistics	Section S8; Table S12
Computational cost and point density	Sections S9–S10; Tables S13–S14; Fig. S5
Training ablations	Section S11; Table S15
Large-pose registration stress test	Section S12; Tables S16–S17; Figs. S6–S7

S1. SurF+XYZ point-wise representation and normalization

For each point $\mathbf{p}_i$, the SurF extractor identifies K=8 nearest neighbors within the same point-cloud batch, orders them by the azimuthal angle $\phi_{ik}=atan2\left(y_{ik}-y_i,x_{ik}-x_i\right)$ in the normalized coordinate frame, and forms eight triangular patches $\left(\mathbf{p}_i,\mathbf{v}_{i,k},\mathbf{v}_{i,k+1}\right)$. Each patch is represented by a 10-dimensional descriptor comprising centroid position (3), triangle normal (3), centroid spherical coordinates (3), and the centroid-normal projection (1). A shared three-layer MLP with hidden dimensions 96 and 96 maps each descriptor to a 10-dimensional feature, and max pooling over patches yields the local SurF vector. Concatenation with normalized XYZ produces a 13-dimensional point-wise input.

For each sample, $\mathbf{p}_{norm}=(\mathbf{p}-\boldsymbol{\mu})s$ and predictions are returned to metric space using $\mathbf{p}=\mathbf{p}_{norm}/s+\boldsymbol{\mu}$. The same sample-specific scale is used to convert inter-expert discrepancies and landmark errors to millimeters. Epsilon-stabilized norms, clipped inverse-cosine inputs, bounded descriptors, and NaN/Inf replacement are applied before MLP processing.

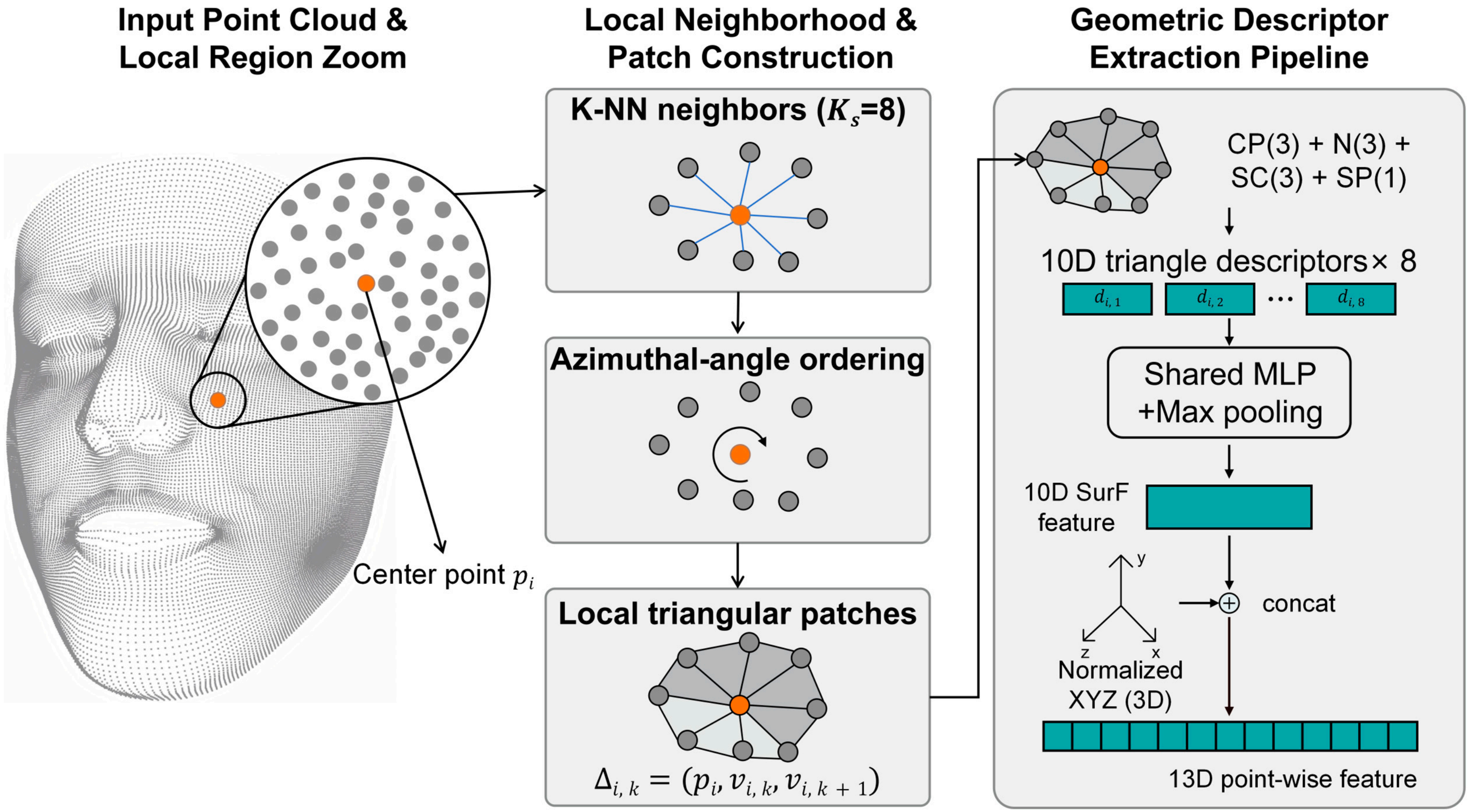

Figure S1: SurF+XYZ point-wise feature construction.

S2. Network, training, and loss settings

Table S2: Implementation and training settings extracted from the archived project configuration and model definitions.

Component	Parameter	Value
Input	Dataset/split	FaceScape; subject-independent 13,520/1,690/1,690 training/validation/test samples
Input	Points/landmarks	16,384 points; bilateral outer canthi, nasal tip, bilateral lower nasolabial fold points
SurF	Local descriptor	K = 8, output d = 10, hidden dimensions (96,96), concatenated XYZ, 13 input channels
Backbone	SLG-GCN	6 layers, 96 hidden channels, KNN size 32, 5 landmark channels
Heatmap	Gaussian width	distance $\sigma = 0.03$; protocol visibility threshold $3\sigma = 0.09$ normalized units
Clean expert	Coordinate decoder	graph-local heatmap peaks; top 8 by heatmap response; peak softmax temperature 0.05; 32-nearest-point feature pooling with distance-softmax temperature 0.10
Clean expert	Training	300 epochs; no occlusion/rotation/noise augmentation; Adam 6×10^{-4}, weight decay 10^{-5}, betas (0.9,0.999), batch 8, gradient clipping 0.5
Clean expert	Loss weights	heatmap 1.0, refined coordinate 14.0, coarse coordinate 2.0, refinement stability 0.02
Occlusion expert	Principal coordinate decoder/prior	all-point heatmap softmax, temperature 1.0; landmark/global context MLP; local offset regression; global token + landmark-identity prior; minimum local weight 0.05
Occlusion expert	Auxiliary peak branch	graph-local top 8, peak temperature 0.08; 32-nearest-point pooling, temperature 0.15; used only by the visible-landmark auxiliary peak-refinement loss
Occlusion expert	Training	300 epochs; surface-loss augmentation probability 0.85 with a 20–35% ratio; rotation probability 0.40, ±30°; Gaussian noise probability 0.40, SD 0.003; Adam 4×10^{-4}, weight decay 2×10^{-5}, betas (0.9,0.999), batch 8, gradient clipping 0.5
Occlusion expert	Loss weights	heatmap 0.20, coordinate 5.0, occluded-coordinate 4.0, prior 2.0, visibility 1.0/2.5, registration 0.2, visible-landmark auxiliary peak-refinement term 0.2, structural consistency 0.4
Final automatic fusion	Scene gate	$q_{0.1}(v) \geq 0.95$, mean gap ≤ 2.0 mm, mean $\sigma \leq 0.042078$
Final automatic fusion	Residual	$\alpha = 0.80$, discrepancy threshold 7.5 mm, nasal-tip residual excluded

S2.1. Clean-expert peak-based coarse-to-fine decoding

For landmark k, let $\mathrm{h}_{ik}^{\mathrm{clean}}$ be the Clean-expert point-wise heatmap response, let $\mathbf{f}_i^{\mathrm{clean}}$ be the corresponding point feature, and let $N(i)$ denote the local KNN-graph neighbors of point i. The sparse implementation used in the reported experiments first identifies graph-local peaks

$$P_k^{\mathrm{clean}}=\left\{i:\mathrm{h}_{ik}^{\mathrm{clean}}\geq\mathrm{h}_{jk}^{\mathrm{clean}},\forall j\in N(i)\right\}.$$

The eight peaks with the largest heatmap responses are retained. Thus, the peak candidates are ranked by heatmap response, not by Euclidean distance. For retained indices i_j, the normalized decoding weights are the temperature-softmax

$$\beta_{kj}=\frac{exp\left(\mathrm{h}_{i_jk}^{\mathrm{clean}}/0.05\right)}{\sum_{r=1}^{8} exp\left(\mathrm{h}_{i_rk}^{\mathrm{clean}}/0.05\right)},$$

$$\mathbf{c}_k^{\mathrm{clean}}=\sum_{j=1}^{8}\beta_{kj}\,\mathbf{p}_{i_j}.$$

After coordinate decoding, Euclidean distance is used in a separate feature-pooling step. The 32 points nearest to $\mathbf{c}_k^{\mathrm{clean}}$ are selected and weighted by

$$\gamma_{kn}=\frac{exp\left(-\|\mathbf{p}_n-\mathbf{c}_k^{\mathrm{clean}}\|_2/0.10\right)}{\sum_{m\square N_k^{32}} exp\left(-\|\mathbf{p}_m-\mathbf{c}_k^{\mathrm{clean}}\|_2/0.10\right)}.$$

Their point features form $\mathbf{u}_k^{\mathrm{clean}}=\sum_n\gamma_{kn}\,\mathbf{f}_n^{\mathrm{clean}}$. The local token, a global mean feature token, and $\mathbf{c}_k^{\mathrm{clean}}$ are concatenated and passed through a refinement MLP. The resulting coordinate is $\hat{\mathbf{l}}_k^{\mathrm{clean}}=\mathbf{c}_k^{\mathrm{clean}}+\Delta\mathbf{l}_k^{\mathrm{clean}}$.. The implementation keys hard_coords_0 and hard_coords correspond, respectively, to the peak-decoded coarse coordinate and refined peak-based coordinate; the manuscript uses these descriptive terms instead of the ambiguous implementation shorthand “hard coordinate.”

S2.2. Occlusion-expert local and global-prior coordinate branches

The principal Occlusion-expert coordinate is not produced by its auxiliary peak branch. Let $\mathrm{h}_{ik}^{\mathrm{occ}}$ and $\mathbf{f}_i^{\mathrm{occ}}$ denote the Occlusion-expert heatmap response and point feature, respectively. A standard all-point heatmap softmax with temperature 1.0 produces

$$\omega_{ik}^{\mathrm{occ}}=\frac{exp(\mathrm{h}_{ik}^{\mathrm{occ}})}{\sum_n exp\,(\mathrm{h}_{nk}^{\mathrm{occ}})},$$

$$\mathrm{c}_k^{\mathrm{soft}}=\sum_i\omega_{ik}^{\mathrm{occ}}\,\mathrm{p}_i,$$

$$\mathbf{z}_k^{occ}=\sum_i\omega_{ik}^{occ}\,\mathbf{f}_i^{occ}.$$

The landmark-specific token $\mathbf{z}_k^{\text{occ}}$ is concatenated with a global mean face token and transformed by a context MLP. A coordinate-regression MLP predicts $\Delta\mathbf{l}_k^{\text{loc}}$, giving $\hat{\mathbf{l}}_k^{\text{loc}}=\mathbf{c}_k^{\text{soft}}+\Delta\mathbf{l}_k^{\text{loc}}$. Independently, the global-prior branch concatenates the global face token with a learned landmark-identity embedding and predicts $\hat{l}_k^{\text{prior}}$. The final principal coordinate is

$$\hat{\mathbf{l}}_k^{\text{occ}}=\tilde{v}_k\hat{\mathbf{l}}_k^{\text{loc}}+(1\text{-}\tilde{v}_k)\hat{\mathbf{l}}_k^{\text{prior}},$$
$$v_k=sigmoid(a_k),$$
$$\tilde{v}_k=\max_{(v_k,0.05)}.$$

The raw v_k, rather than $\tilde{v}_k$, is retained for visibility calibration and scene gating. The separate graph-local peak branch uses eight peaks, temperature 0.08, and 32-nearest-point pooling with temperature 0.15; it contributes only through the visible-landmark auxiliary peak-refinement loss and is not the formal pred_coords output.

S2.3. Complete training objectives and masking definitions

The heatmap term for both experts is the Adaptive Wing heatmap loss used by the implementation, denoted by $\mathrm{L}_{\mathrm{h}m}$, with ω=14 and θ=0.5. For a landmark mask $m_k\in\{0,1\}$, define the masked coordinate loss

$$\mathrm{L}_{\mathrm{L}1}(\mathbf{x},\mathbf{y};\mathbf{m})=\frac{\sum_k m_k\frac{1}{3}\left\|\mathbf{x}_k\text{-}\mathbf{y}_k\right\|_1}{\sum_k m_k+\varepsilon}.$$

Let $r_k\in\{0,1\}$ denote protocol visibility and o_k=1-r_k denote protocol-missing status. The Clean-expert objective is

$$\mathrm{L}_{clean}\ =1.0\mathrm{L}_{\mathrm{h}m}+14.0\mathrm{L}_{L1}\left(\hat{\mathbf{l}}^{clean},\mathbf{l};\mathbf{r}\right)+2.0\mathrm{L}_{L1}\left(\mathbf{c}^{clean},\mathbf{l};\mathbf{r}\right)+0.02\mathrm{L}_{L1}\left(\hat{\mathbf{l}}^{clean},\mathbf{c}^{clean};\mathbf{r}\right),$$

where $\mathbf{c}^{\text{clean}}$ is the initial peak-decoded coordinate and $\hat{\mathbf{l}}^{\text{clean}}$ is the refined coordinate. For the Occlusion expert, the raw visibility probability is $v_k=sigmoid(a_k)$ and the internal local-prior coordinate fusion uses $\tilde{v}_k=\max(v_k,0.05)$. The raw v_k is retained for calibration and automatic scene gating. The uncertainty head produces an unconstrained log-variance proxy s_k; the clipped variable used in the training objective is

$$\bar{s}_k=clip(s_k,\text{-}6,2).$$

The all-landmark heteroscedastic coordinate term is

$$\mathrm{L}_{\text{het}}=\frac{1}{5}\sum_{k=1}^{5}\left[exp(\text{-}\bar{s}_k)\mathrm{l}_{\text{SmoothL1}}\left(\hat{\mathbf{l}}_k^{\text{occ}},\mathbf{l}_k\right)+\bar{s}_k\right],\quad \sigma_k=exp(s_k/2).$$

The raw-output scale σ_k is used only as a relative reliability proxy. The protocol-missing coordinate and prior-coordinate terms are

$$\mathrm{L}_{\text{occ}}=\mathrm{L}_{\mathrm{L}1}\left(\hat{\mathbf{l}}^{\text{occ}},\mathbf{l};\mathbf{o}\right),$$
$$\mathrm{L}_{\text{prior}}=\mathrm{L}_{\mathrm{L}1}\left(\hat{\mathbf{l}}^{\text{prior}},\mathbf{l};\mathbf{o}\right).$$

The balanced visibility term is

$$\mathrm{L}_{\mathrm{vis}}=\frac{\sum_k[r_k w_{\mathrm{vis}}+(1\text{-}r_k)w_{\mathrm{miss}}]\ \mathrm{BCEWithLogits}(a_k,r_k)}{\sum_k[r_k w_{\mathrm{vis}}+(1\text{-}r_k)w_{\mathrm{miss}}]+\varepsilon},\quad w_{\mathrm{vis}}=1.0,\quad w_{\mathrm{miss}}=2.5.$$

For the structural term, define

$$D_{ij}(\mathbf{L})=\sqrt{\left\|\mathbf{l}_i\text{-}\mathbf{l}_j\right\|_2^2+\varepsilon},\quad P=\{(i,j):i\neq j,o_i+o_j>0\}.$$

Then

$$\mathrm{L}_{\mathrm{struct}}=\frac{1}{|P|}\sum_{(i,j)\square P}\mathrm{SmoothL1}\left(D_{ij}\left(\widehat{\mathbf{L}}^{occ}\right),D_{ij}(\mathbf{L})\right),$$

with zero loss when P is empty. The visible-landmark auxiliary peak-refinement term (implementation key w_hard) is

$$\mathrm{L}_{\mathrm{peak}}=\mathrm{L}_{\mathrm{L1}}\left(\hat{\mathbf{l}}^{\mathrm{peak}},\mathbf{l};\mathbf{r}\right).$$

During training, the auxiliary registration coupling estimates a weighted Procrustes transform $(\widehat{\mathbf{R}},\hat{\mathbf{t}})$ from paired predicted landmarks A and B using

$$w_k=v_k^A v_k^B exp\left(\text{-}\bar{s}_k^A\right)exp\left(\text{-}\bar{s}_k^B\right),$$

where the exponential factors are clipped precision-like weights. The transform is then applied to the ground-truth landmarks of sample A and supervised against sample B:

$$\mathrm{L}_{\mathrm{reg}}=Smooth\mathrm{h}L1\left(\mathbf{L}^A\widehat{\mathbf{R}}^{\mathsf{T}}+\hat{\mathbf{t}},\mathbf{L}^B\right).$$

The complete Occlusion-expert objective is

$$\mathrm{L}_{occ\ expert}\quad=0.20\mathrm{L}_{\mathrm{h}m}+5.0\mathrm{L}_{\mathrm{h}et}+4.0\mathrm{L}_{occ}+2.0\mathrm{L}_{prior}+\mathrm{L}_{vis}+0.4\mathrm{L}_{struct}+0.2\mathrm{L}_{peak}+0.2\mathrm{L}_{reg}.$$

The configured coarse peak-coordinate and peak-refinement-stability compatibility weights for the Occlusion expert are zero, so those compatibility terms do not contribute to the reported training objective.

S3. Controlled evaluation protocols

The primary analysis uses 0%, 30%, and 50% controlled spherical local surface loss because complete per-landmark expert outputs were archived at those levels, allowing the final automatic gate and all alternative fusion rules to be evaluated consistently. Structured testing uses twelve patterns at 30% and 50% intensity. Each condition contains 1,690 test samples and 8,450 landmark evaluations.

S4. Validation-only selection of automatic gating and residual fusion

The final parameter configuration was selected exclusively from validation-set outputs; test-set performance was not used to rank the candidate configurations. The lower-tail visibility threshold was searched over {0.4, 0.5, 0.6, 0.7, 0.8, 0.9, 0.95}, the mean scene-gap threshold over {0.5, 1, 1.5, 2, 3, 4, 5, 7.5, 10} mm, and the mean-uncertainty threshold over the 0.50, 0.60, 0.70, 0.80, 0.90, and 0.95 validation quantiles plus the validation maximum. The scene-gate objective minimized validation sample-level mean error, then the > 6 mm sample failure rate, and then the Clean-gate rate. $Q_{0.1}$ was computed using the default linear-interpolation quantile definition in NumPy/PyTorch. The residual grid used $\alpha\in\{0,0.2,0.4,0.6,0.8,1.0\}$, discrepancy thresholds {1, 2, 3, 4, 5, 7.5, 10} mm, and

either no landmark exclusion or exclusion of each of the five landmarks. Selection minimized validation sample-level mean error on the occluded validation scenes, followed by the > 6 mm failure rate and deterministic tie breakers defined in the analysis script. The selected combination was α=0.80, 7.5 mm, with nasal-tip exclusion; it was recovered in 200/200 bootstrap selections.

Table S3: Original and validation-selected residual settings on the test set.

Setting	0% (mm)	30% (mm)	50% (mm)	Aggregate (mm)
Original protocol-stratified rule, α = 0.40, 3 mm	0.467	1.205	2.473	1.381
Validation-selected protocol-stratified rule, α = 0.80, 7.5 mm	0.467	1.146	2.216	1.276
Final automatic scene gate + selected residual	0.470	1.159	2.216	1.281

Aggregate values were calculated from unrounded condition-level means and may therefore differ by 0.001 mm from arithmetic based on the displayed rounded values.

Condition	Final error (mm)	Clean-scene gate rate	Agreement with protocol-defined group
0% loss	0.470 ± 0.688	98.22%	98.22%
30% loss	1.159 ± 1.093	3.96%	96.04%
50% loss	2.216 ± 2.169	0.00%	100.00%

Table S4: Automatic scene-gate behavior on the independent test set.

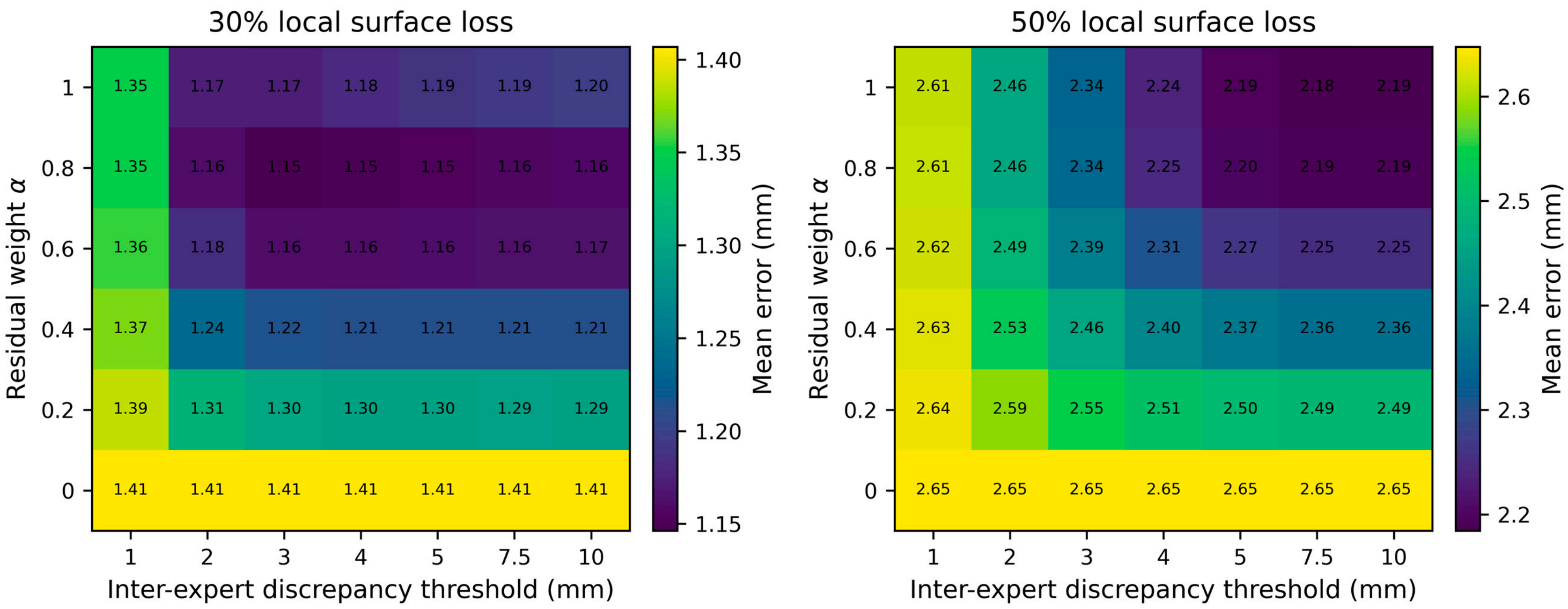

Figure S2: Validation sensitivity surfaces at 30% and 50% loss with nasal-tip exclusion.

Table S5: Full primary localization summaries at 0%, 30%, and 50% controlled local surface loss. Failure rates are calculated over 8,450 landmarks per condition. Unified OASR uses pred_coords.

Loss	Method	n	Mean ± SD (mm)	Median (mm)	> 2 mm (%)	> 4 mm (%)	> 6 mm (%)
0%	2S-SGCN baseline	8,450	1.114 ± 0.763	0.912	13.08	0.39	0.05
0%	PointNet++ baseline	8,450	0.665 ± 0.606	0.525	2.40	0.13	0.05
0%	Unified OASR	8,450	1.006 ± 0.683	0.898	4.43	0.39	0.12
0%	Clean expert	8,450	0.467 ± 0.649	0.314	2.05	0.12	0.05
0%	Occlusion expert	8,450	1.254 ± 0.710	1.175	9.09	0.28	0.11
0%	Final automatic dual expert	8,450	0.470 ± 0.688	0.317	1.96	0.13	0.07
30%	2S-SGCN baseline	8,450	15.185 ± 31.282	1.473	36.08	21.49	20.30
30%	PointNet++ baseline	8,450	12.448 ± 26.157	0.677	24.27	21.08	20.22
30%	Unified OASR	8,450	1.816 ± 1.858	1.276	25.57	8.54	3.36
30%	Clean expert	8,450	12.414 ± 23.902	1.089	30.69	21.82	20.90
30%	Occlusion expert	8,450	1.394 ± 0.979	1.203	14.79	2.12	0.43
30%	Final automatic dual expert	8,450	1.159 ± 1.093	0.858	14.27	2.40	0.46
50%	2S-SGCN baseline	8,450	28.212 ± 39.489	2.784	60.52	45.24	43.30
50%	PointNet++ baseline	8,450	27.276 ± 35.049	8.588	47.51	45.10	43.48
50%	Unified OASR	8,450	4.626 ± 4.209	3.424	76.44	41.79	23.74
50%	Clean expert	8,450	27.598 ± 34.075	2.139	50.71	46.31	45.09
50%	Occlusion expert	8,450	2.669 ± 1.982	2.119	53.09	19.11	6.56
50%	Final automatic dual expert	8,450	2.216 ± 2.169	1.437	39.81	17.35	6.63

S5. Calibration and landmark-wise behavior

Table S6: Visibility calibration and uncertainty–error association.

Group	n	Accuracy	Balanced acc.	AUROC	AUPRC	Brier	ECE	Spearman ρ
All	25,350	98.87%	98.44%	0.99895	0.99979	0.00862	0.00486	0.092
0%*	8,450	100.00%	100.00%	–	1.00000	0.00000	0.00040	0.110

Group	n	Accuracy	Balanced acc.	AUROC	AUPRC	Brier	ECE	Spearman ρ
30%	8,450	99.33%	98.99%	0.99920	0.99980	0.00565	0.00295	0.147
50%	8,450	97.29%	97.33%	0.99718	0.99875	0.02020	0.01257	0.334

** The 0% condition contains only visible labels; class-balanced discrimination metrics are therefore not informative for this single-class group.*

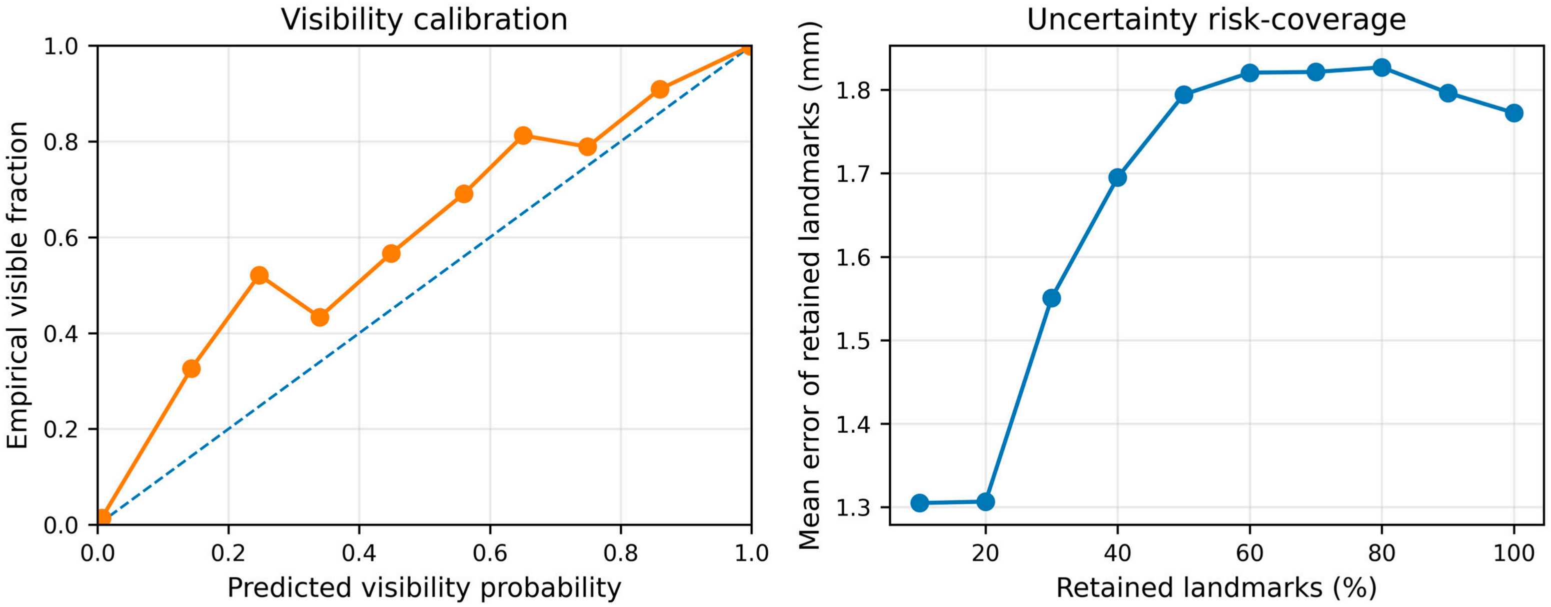

Figure S3: Visibility calibration and uncertainty risk-coverage diagnostics. Visibility showed high classification performance and low aggregate calibration error for the controlled synthetic labels, whereas uncertainty was only weakly associated with localization error.

Table S7: Final automatic method by landmark and protocol-defined visibility. N visible/missing reports the subset sizes; all errors are in millimeters.

Loss	Landmark	N visible/missing	Overall mean ± SD	Median	Visible mean	Missing mean	> 6 mm (%)
30%	Right outer canthus	1391/299	1.003 ± 1.102	0.729	0.780	2.038	0.18
30%	Left outer canthus	1368/322	0.873 ± 0.789	0.631	0.678	1.705	0.06
30%	Nasal tip	1343/347	1.442 ± 0.917	1.263	1.307	1.965	0.36
30%	Right lower nasolabial fold point	1461/229	1.312 ± 1.201	0.941	1.015	3.209	0.89
30%	Left lower nasolabial fold point	1498/192	1.162 ± 1.281	0.742	0.881	3.352	0.83
30%	All landmarks	7061/1389	1.159 ± 1.093	0.858	0.931	2.317	0.46
50%	Right outer canthus	1321/369	1.543 ± 1.867	1.021	1.035	3.358	2.96
50%	Left outer canthus	1296/394	1.196 ± 1.315	0.743	0.779	2.568	1.48
50%	Nasal tip	859/831	3.200 ± 1.701	2.948	3.149	3.253	6.63
50%	Right lower nasolabial fold point	1178/512	2.560 ± 2.418	1.564	1.479	5.046	10.41
50%	Left lower nasolabial fold point	1178/512	2.584 ± 2.619	1.409	1.446	5.200	11.66
50%	All landmarks	5832/2618	2.216 ± 2.169	1.437	1.462	3.896	6.63

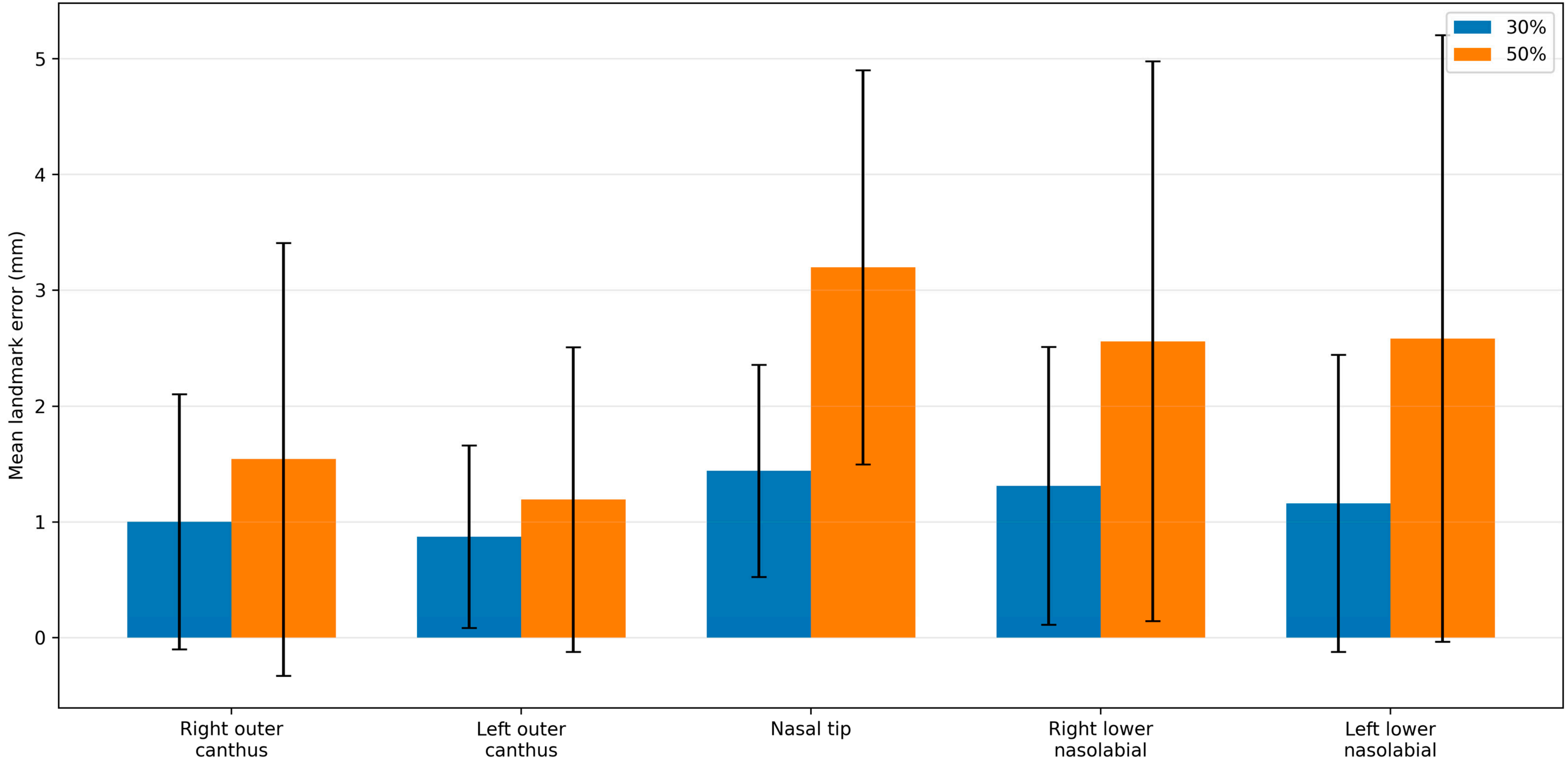

Figure S4: Landmark-wise mean errors for the final automatic method at 30% and 50% loss.

S6. Stronger fusion baselines

Table S8: Full fusion-strategy comparison. Errors are mean millimeters.

Strategy	0%	30%	50%	All
Unified OASR	1.006	1.816	4.626	2.483
Occ-only	1.254	1.394	2.669	1.772

Strategy	0%	30%	50%	All
Simple average	0.741	6.525	14.123	7.130
Visibility hard routing	0.467	3.080	8.031	3.859
Uncertainty-weighted soft fusion	0.610	6.342	9.190	5.381
Uncertainty-only learned hard router	0.611	6.740	6.837	4.729
Learned MoE soft router	0.538	1.142	2.245	1.308
Learned MoE hard router	0.515	1.161	2.222	1.299
Learned residual gate	0.528	1.405	2.777	1.570
Best-expert oracle	0.404	1.053	2.126	1.194
Final automatic dual expert	0.470	1.159	2.216	1.281

Aggregate values were calculated from unrounded condition-level means and may therefore differ by 0.001 mm from arithmetic based on the displayed rounded values.

The visibility hard-routing row uses predicted visibility probabilities with a threshold of 0.5 and is therefore an inference-time strategy rather than an oracle. The best-expert oracle selects, for each landmark, the expert with the smaller ground-truth localization error and is reported only as a ground-truth-dependent upper bound.

Table S9: Detailed fusion diagnostics at 30% controlled local surface loss. Mean Clean weight is the average coordinate weight assigned to the Clean expert.

Strategy	Mean ± SD (mm)	Visible mean	Missing mean	> 6 mm (%)	Mean Clean weight
Clean-only	12.414 ± 23.902	3.332	58.578	20.90	1.000
Occ-only	1.394 ± 0.979	1.212	2.317	0.43	0.000
Simple average	6.525 ± 11.839	2.038	29.333	20.32	0.500
Visibility hard routing	3.080 ± 9.864	3.089	3.035	4.59	0.834
Uncertainty-weighted soft fusion	6.342 ± 13.402	1.762	29.624	18.85	0.500
Uncertainty-only learned hard router	6.740 ± 18.867	1.646	32.640	9.03	0.539
Learned MoE soft router	1.142 ± 1.096	0.910	2.321	0.46	0.476
Learned MoE hard router	1.161 ± 1.102	0.934	2.317	0.47	0.557
Learned residual gate	1.405 ± 1.698	1.026	3.328	3.53	0.538
Final automatic dual expert	1.159 ± 1.093	0.931	2.317	0.46	0.532
Best-expert oracle	1.053 ± 1.049	0.804	2.317	0.38	0.483

Table S10: Detailed fusion diagnostics at 50% controlled local surface loss.

Strategy	Mean ± SD (mm)	Visible mean	Missing mean	> 6 mm (%)	Mean Clean weight
Clean-only	27.598 ± 34.075	10.432	65.840	45.09	1.000
Occ-only	2.669 ± 1.982	2.118	3.896	6.56	0.000
Simple average	14.123 ± 16.749	5.785	32.698	43.49	0.500
Visibility hard routing	8.031 ± 19.197	9.101	5.647	18.60	0.679
Uncertainty-weighted soft fusion	9.190 ± 13.828	3.021	22.931	34.53	0.425
Uncertainty-only learned hard router	6.837 ± 18.574	1.964	17.691	11.78	0.395
Learned MoE soft router	2.245 ± 2.190	1.505	3.896	6.97	0.389
Learned MoE hard router	2.222 ± 2.188	1.470	3.896	6.54	0.461
Learned residual gate	2.777 ± 3.148	1.698	5.180	13.21	0.420
Final automatic dual expert	2.216 ± 2.169	1.462	3.896	6.63	0.420
Best-expert oracle	2.126 ± 2.180	1.331	3.896	6.44	0.434

S7. Structured and sensor-inspired synthetic corruptions

Table S11: Pattern-wise results for final automatic fusion versus Unified OASR. Difference is final minus Unified; negative values favor the final method.

Pattern	Intensity	Final (mm)	Unified (mm)	Difference (mm)	Clean-gate rate
Global random reduction	30%	0.776	1.096	-0.320	91.30%
Global random reduction	50%	1.228	1.290	-0.062	45.15%
Contiguous random loss	30%	1.104	1.804	-0.701	3.55%
Contiguous random loss	50%	2.166	4.612	-2.445	0.00%
Left-side truncation	30%	2.401	7.806	-5.406	0.00%
Left-side truncation	50%	3.304	11.645	-8.341	0.00%
Right-side truncation	30%	2.044	8.442	-6.399	0.00%
Right-side truncation	50%	3.044	11.723	-8.679	0.00%
Vertical stripe	30%	1.252	1.439	-0.187	58.70%
Vertical stripe	50%	1.953	1.997	-0.044	11.01%

Pattern	Intensity	Final (mm)	Unified (mm)	Difference (mm)	Clean-gate rate
Horizontal stripe	30%	1.595	2.162	-0.567	41.24%
Horizontal stripe	50%	2.760	3.561	-0.802	16.51%
Non-uniform depth loss	30%	0.961	1.538	-0.577	0.00%
Non-uniform depth loss	50%	1.491	2.774	-1.284	0.00%
Nasal-tip targeted loss	30%	0.880	1.558	-0.678	0.00%
Nasal-tip targeted loss	50%	1.112	2.306	-1.194	0.00%
Right-eye targeted loss	30%	0.936	1.540	-0.605	0.00%
Right-eye targeted loss	50%	1.379	3.563	-2.184	0.00%
Left-eye targeted loss	30%	0.957	1.724	-0.768	0.00%
Left-eye targeted loss	50%	1.486	3.013	-1.527	0.00%
Local loss + quantization	30%	1.275	1.788	-0.514	3.43%
Local loss + quantization	50%	2.313	4.634	-2.321	0.00%
Mixed sensor-inspired	30%	1.569	1.482	0.087	33.08%
Mixed sensor-inspired	50%	2.273	2.051	0.222	3.31%

The final method was better in 22/24 conditions. The two exceptions were the mixed sensor-inspired perturbation at 30% and 50%, where Unified OASR was better by 0.087 and 0.222 mm. These experiments evaluate controlled synthetic perturbations and do not constitute validation on real structured-light or depth-camera data.

S8. Paired statistical comparisons

Table S12: Paired sample-level comparisons for the final automatic method. Positive improvement favors the final method; intervals are paired bootstrap 95% confidence intervals over 1,690 test samples.

Loss	Comparator	Mean improvement (mm)	95% bootstrap CI	Final better	Holm p
30%	Occ-only	0.236	[0.224, 0.247]	86.63%	< 0.001
30%	Unified OASR	0.658	[0.618, 0.698]	83.96%	< 0.001
50%	Occ-only	0.452	[0.437, 0.468]	93.20%	< 0.001
50%	Unified OASR	2.409	[2.271, 2.550]	85.21%	< 0.001

S9. Computational cost

Table S13: Model size, latency, and GPU memory on 16,384-point inputs.

Method	Parameters	Checkpoint MB	Mean latency ms	P95 latency ms	Peak GPU MB
Clean expert	302,612	3.16	158.4 ± 1.5	161.0	444.8
Occlusion expert	393,786	4.33	158.1 ± 1.8	160.7	445.1
2S-SGCN baseline	206,309	2.14	131.3 ± 1.1	133.0	444.0
Unified OASR	323,863	3.12	162.3 ± 1.9	165.9	444.9
Dual expert (sequential)	696,398	7.49	225.7 ± 1.6	228.2	447.4

S10. Point-density sensitivity

Table S14: Final automatic inference across input point counts. Density experiments were rerun independently with density-specific resampling and a fixed random seed; rounded 16,384-point values may therefore differ from the primary evaluation by approximately 0.001 mm.

Points	Loss	Mean error	SD	> 6 mm
4,096	0%	0.469	0.688	0.07%
4,096	30%	1.160	1.093	0.46%
4,096	50%	2.217	2.169	6.64%
8,192	0%	0.469	0.688	0.07%
8,192	30%	1.159	1.093	0.46%
8,192	50%	2.217	2.169	6.63%
16,384	0%	0.470	0.688	0.07%
16,384	30%	1.159	1.093	0.46%
16,384	50%	2.217	2.169	6.63%

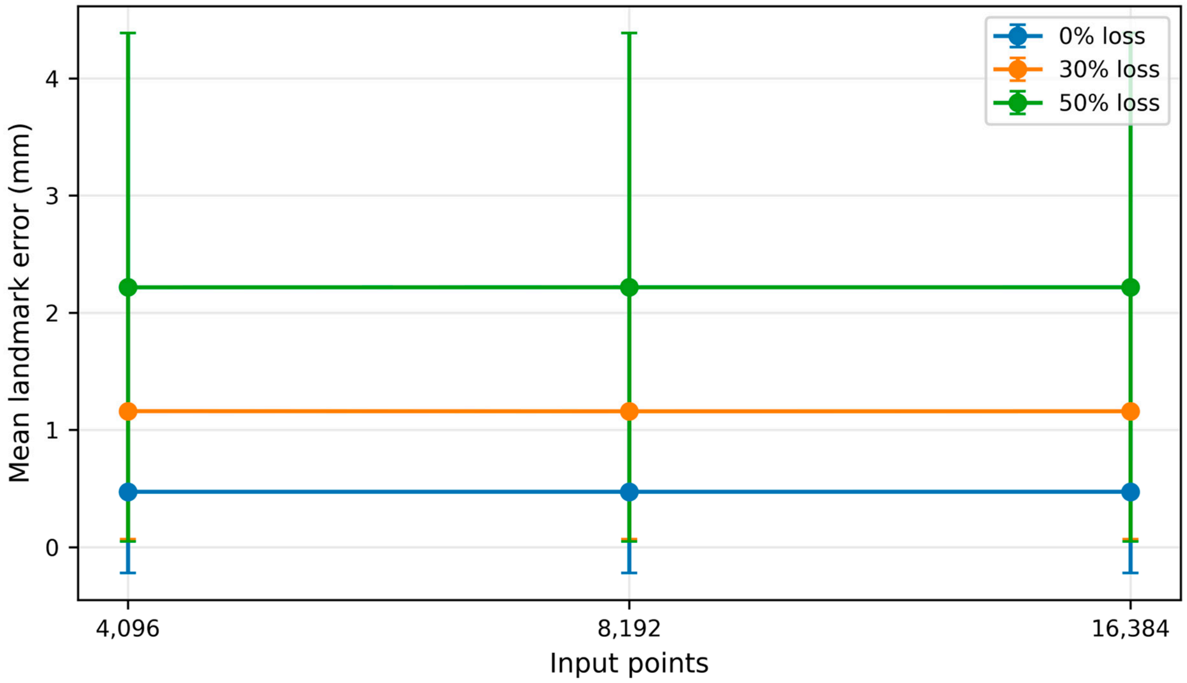

Figure S5: Point-density sensitivity within the controlled FaceScape protocol.

S11. Training-design ablations

Table S15: Occlusion-expert training ablations. The full-model row is the within-ablation reference.

Variant	30% mean ± SD	50% mean ± SD	30% visible	30% missing	30% > 6 mm	Vis. acc.
Full model (ablation run)	1.221 ± 1.052	2.470 ± 2.115	0.990	2.358	0.53%	98.95%
Without occlusion-specific training	13.817 ± 5.456	22.234 ± 7.125	12.844	18.615	94.78%	83.14%
Without visibility supervision	1.263 ± 0.995	2.002 ± 1.659	1.069	2.224	0.30%	16.86%
Without global prior branch	1.686 ± 1.352	2.719 ± 3.119	1.460	2.801	1.80%	98.97%
Without occluded-coordinate emphasis	1.530 ± 1.299	2.595 ± 1.983	1.251	2.903	1.55%	98.88%
Without structural consistency	1.747 ± 1.389	2.997 ± 2.015	1.462	3.152	1.92%	98.70%

These ablations support the use of occlusion-specific training, the global prior, occluded-coordinate emphasis, and structural consistency. The visibility-supervision ablation also shows that low localization error can coexist with unusable visibility estimates, which matters for the automatic scene gate.

S12. Large-pose registration stress test

The stress test used 1,690 samples, five trials per sample, a 180° x-axis flip, base translation (80, 80, 120) mm, and ±10 mm translation jitter. ICP success required final rotation error below 3° and translation error below 3 mm. The dual-expert predictions used the same final automatic gate, residual weight α=0.80, 7.5 mm discrepancy threshold, and nasal-tip residual exclusion as the primary localization analysis. The 30% ratio generated the controlled moving surfaces but was not passed to fusion. The original random perturbation transforms were reused exactly. Descriptive values below use 8,450 trials; paired tests use trial-averaged outcomes for 1,690 independent test samples.

Table S16: Large-pose stress-test results using final automatic dual-expert inference.

Initialization	Coarse rotation	Coarse translation	Pre-ICP RMSE	ICP success	Final ICP RMSE
Direct ICP	–	–	68.364 ± 7.051	0.05%	68.807 ± 125.940
Reference landmarks	0.000 ± 0.000	0.000 ± 0.000	0.446 ± 0.076	100.00%	0.446 ± 0.076
Occlusion expert	0.876 ± 0.641	1.949 ± 1.593	1.084 ± 0.400	100.00%	0.446 ± 0.076
2S-SGCN baseline	18.711 ± 17.303	33.709 ± 27.725	12.024 ± 8.525	98.82%	0.779 ± 3.104
Unified OASR	1.191 ± 0.841	2.271 ± 1.801	1.369 ± 0.518	100.00%	0.446 ± 0.076
Final automatic dual	0.784 ± 0.642	1.696 ± 1.564	0.988 ± 0.430	100.00%	0.446 ± 0.076

Initialization	Coarse rotation	Coarse translation	Pre-ICP RMSE	ICP success	Final ICP RMSE
expert					

Table S17: Sample-level paired comparison of final automatic dual-expert registration outputs. Positive improvement favors the final dual expert. Holm correction was applied across the twelve comparator–metric tests.

Comparator	Metric	n	Comparator	Final dual	Improvement (95% CI)	Holm p
Unified OASR	Coarse rotation (°)	1,690	1.1907	0.7842	0.4065 [0.3729, 0.4406]	< 0.001
Unified OASR	Coarse translation (mm)	1,690	2.2713	1.6960	0.5753 [0.5056, 0.6471]	< 0.001
Unified OASR	Pre-ICP RMSE (mm)	1,690	1.3691	0.9883	0.3808 [0.3586, 0.4030]	< 0.001
Unified OASR	Post-ICP rotation (°)	1,690	0.004515	0.004514	0.000001 [−0.000005, 0.000007]	0.487
Unified OASR	Post-ICP translation (mm)	1,690	0.012609	0.012614	−0.000005 [−0.000025, 0.000015]	1.000
Unified OASR	Post-ICP RMSE (mm)	1,690	0.445826	0.445825	< 0.000001 [−0.000001, 0.000002]	1.000
Occ-only	Coarse rotation (°)	1,690	0.8764	0.7842	0.0922 [0.0783, 0.1063]	< 0.001
Occ-only	Coarse translation (mm)	1,690	1.9487	1.6960	0.2527 [0.2165, 0.2883]	< 0.001
Occ-only	Pre-ICP RMSE (mm)	1,690	1.0836	0.9883	0.0953 [0.0848, 0.1059]	< 0.001
Occ-only	Post-ICP rotation (°)	1,690	0.004517	0.004514	0.000002 [−0.000003, 0.000008]	1.000
Occ-only	Post-ICP translation (mm)	1,690	0.012620	0.012614	0.000006 [−0.000010, 0.000022]	0.953
Occ-only	Post-ICP RMSE (mm)	1,690	0.445826	0.445825	< 0.000001 [−0.000001, 0.000001]	1.000

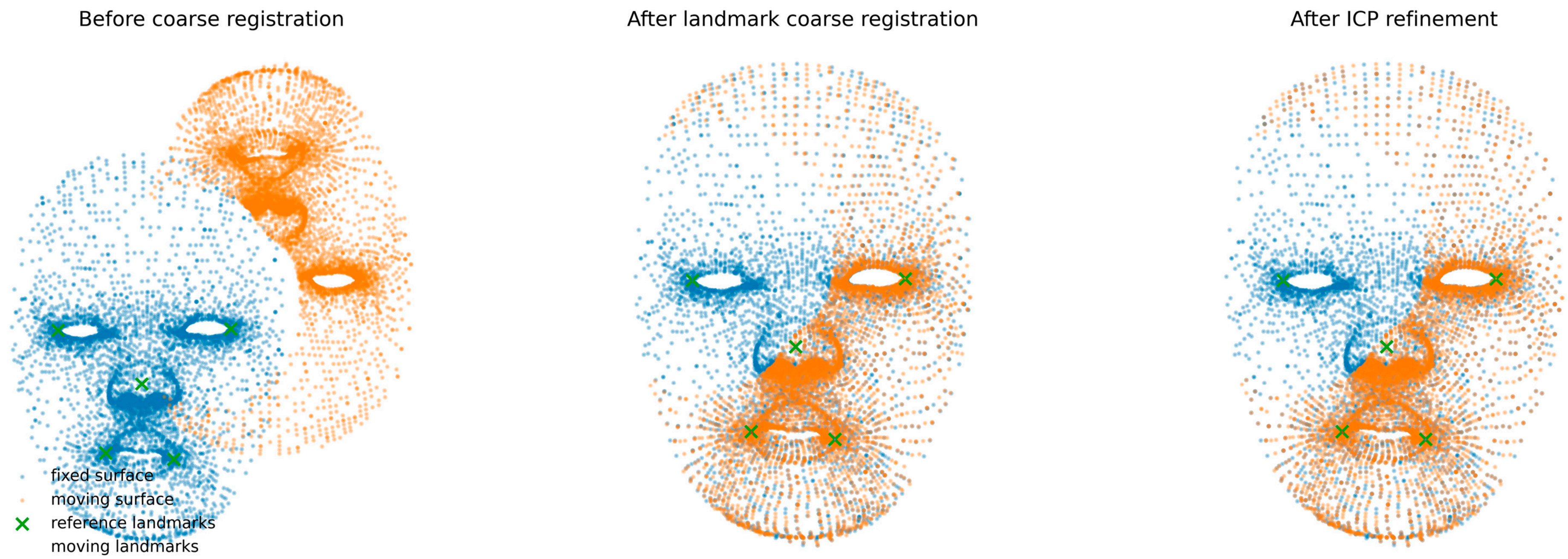

Figure S6: Representative large-pose stress-test projections before coarse initialization, after landmark-based initialization, and after ICP.

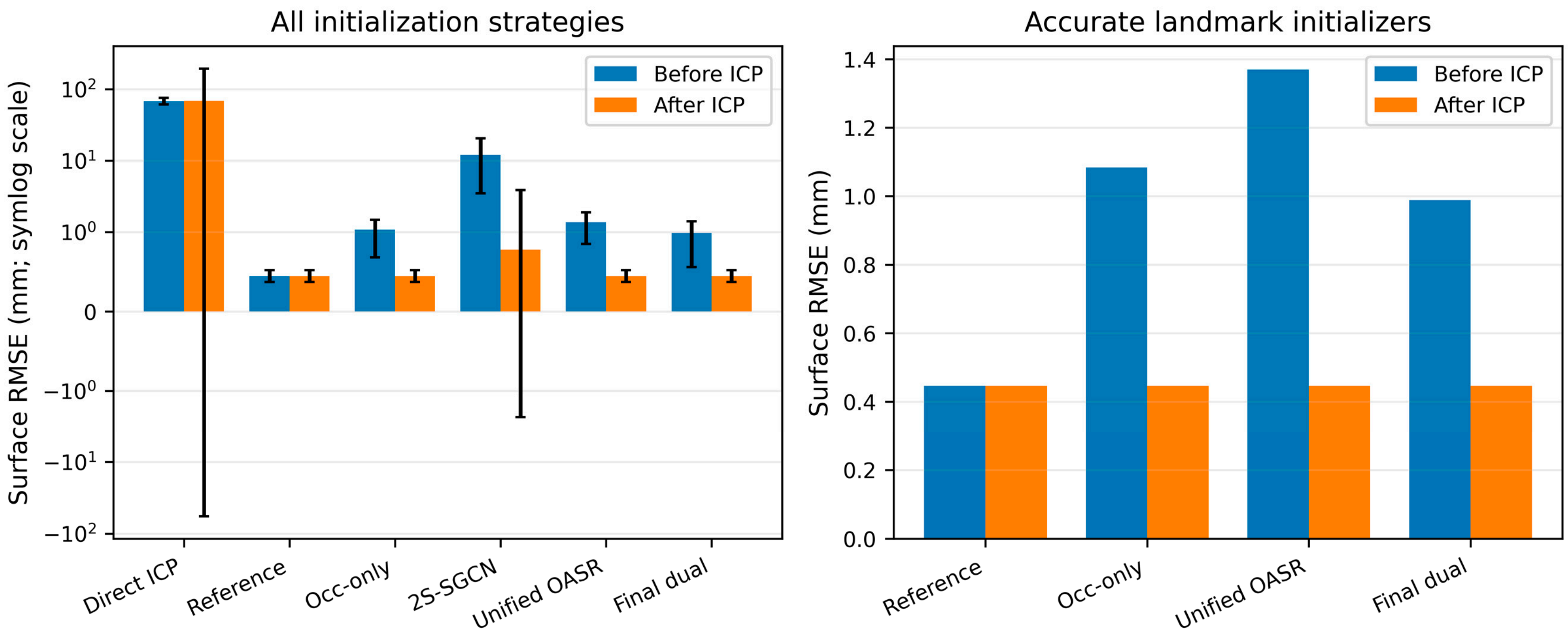

Figure S7: Surface RMSE before and after ICP in the large-pose stress test.
